# Direct transformation of dinitrogen: synthesis of *N*-containing organic compounds via N−C bond formation

**DOI:** 10.1093/nsr/nwaa142

**Published:** 2020-06-23

**Authors:** Ze-Jie Lv, Junnian Wei, Wen-Xiong Zhang, Ping Chen, Dehui Deng, Zhang-Jie Shi, Zhenfeng Xi

**Affiliations:** Beijing National Laboratory for Molecular Sciences (BNLMS), Key Laboratory of Bioorganic Chemistry and Molecular Engineering of Ministry of Education, College of Chemistry, Peking University, Beijing 100871, China; Beijing National Laboratory for Molecular Sciences (BNLMS), Key Laboratory of Bioorganic Chemistry and Molecular Engineering of Ministry of Education, College of Chemistry, Peking University, Beijing 100871, China; Beijing National Laboratory for Molecular Sciences (BNLMS), Key Laboratory of Bioorganic Chemistry and Molecular Engineering of Ministry of Education, College of Chemistry, Peking University, Beijing 100871, China; Dalian Institute of Chemical Physics, Chinese Academy of Sciences, Dalian 116023, China; Dalian Institute of Chemical Physics, Chinese Academy of Sciences, Dalian 116023, China; Department of Chemistry, Fudan University, Shanghai 200433, China; Beijing National Laboratory for Molecular Sciences (BNLMS), Key Laboratory of Bioorganic Chemistry and Molecular Engineering of Ministry of Education, College of Chemistry, Peking University, Beijing 100871, China

**Keywords:** dinitrogen transformation, metal-dinitrogen complex, N−C bond formation, *N*-containing organic compounds

## Abstract

*N*-containing organic compounds are of vital importance to lives. Practical synthesis of valuable *N*-containing organic compounds directly from dinitrogen (N_2_), not through ammonia (NH_3_), is a holy-grail in chemistry and chemical industry. An essential step for this transformation is the functionalization of the activated N_2_ units/ligands to generate N−C bonds. Pioneering works of transition metal-mediated direct conversion of N_2_ into organic compounds via N−C bond formation at metal-dinitrogen [N_2_-M] complexes have generated diversified coordination modes and laid the foundation of understanding for the N−C bond formation mechanism. This review summarizes those major achievements and is organized by the coordination modes of the [N_2_-M] complexes (end-on, side-on, end-on-side-on, etc.) that are involved in the N−C bond formation steps, and each part is arranged in terms of reaction types (*N*-alkylation, *N*-acylation, cycloaddition, insertion, etc.) between [N_2_-M] complexes and carbon-based substrates. Additionally, earlier works on one-pot synthesis of organic compounds from N_2_ via ill-defined intermediates are also briefed. Although almost all of the syntheses of *N*-containing organic compounds via direct transformation of N_2_ so far in the literature are realized in homogeneous stoichiometric thermochemical reaction systems and are discussed here in detail, the sporadically reported syntheses involving photochemical, electrochemical, heterogeneous thermo-catalytic reactions, if any, are also mentioned. This review aims to provide readers with an in-depth understanding of the state-of-the-art and perspectives of future research particularly in direct catalytic and efficient conversion of N_2_ into *N*-containing organic compounds under mild conditions, and to stimulate more research efforts to tackle this long-standing and grand scientific challenge.

## Teaser

This review provides summary and perspectives on direct functionalization/transformation of dinitrogen into N-containing organic compounds, via homogeneous, heterogeneous, thermochemical, photochemical, electrochemical, and other available reaction systems.

## INTRODUCTION

As the most abundant constituent in Earth's atmosphere (atm), dinitrogen (N_2_) is the main nitrogen source of *N*-containing compounds on the Earth. Therefore, N_2_ fixation and activation are essential both for nature and humans. Nevertheless, the high bond dissociation energy (942 kJ/mol) and large highest occupied molecular orbital (HOMO)—lowest unoccupied molecular orbital (LUMO) gap (10.82 eV) make N_2_ exhibit extremely low reactivity and be regarded as an inert gas. Currently, the N_2_ fixation and conversion in nature and industry mainly rely on two pathways, in which ammonia (NH_3_) is the product [1]. In nature, nitrogenase metalloenzymes employ iron-sulfur clusters as the key cofactor (FeMo, FeV or FeFe cofactor) and water as the proton source to transfer N_2_ into NH_3_ at ambient temperature and pressure [2]. This biosynthetic NH_3_ is a versatile precursor for the synthesis of *N*-containing organic compounds, such as amino acids and nucleic acids. Although the precise biological N_2_ reduction mechanism is still controversial, spectroscopic and computational studies suggested the presence of an interstitial carbon atom at the center of the FeMo and FeV cofactors [3–5].

In industry, more than 170 million metric tons of NH_3_ is produced from the Haber-Bosch process annually, in which N_2_ reacts with dihydrogen (H_2_) under the harsh condition in the presence of iron or ruthenium catalysts. This NH_3_ synthesis process consumes 1–2% of the world's annual energy supply along with the huge CO_2_ emission, due to the drastic reaction condition and the energy requirement for H_2_ production from fossil fuels and water [6]. As the main route of N_2_ fixation and transformation in industry, ∼20% of NH_3_ produced from the Haber-Bosch process is used as the feedstock to produce *N*-containing chemicals, including higher-value *N*-containing organic compounds, like amines, nitriles, nitro and so on. To better understand the reaction mechanism of biological and industrial reduction of N_2_ into NH_3_, several catalytic systems including homogeneous molecular systems, electrochemical systems and heterogeneous systems have been studied for decades, and there are comprehensive reviews that readers may refer to [7–13].

Compared to NH_3_-based N_2_ fixation process, an alternative route of N_2_ fixation is the direct conversion of N_2_ into *N*-containing organic compounds under mild condition. This approach is always targeted because it provides the potential solution to developing a sustainable system with reduced fossil fuel requirements. The earliest study towards this goal began in the 1960s, when Vol’pin *et al.* discovered that the titanium species, for example, Cp_2_TiCl_2_ could react with PhLi under N_2_ to give aniline after hydrolysis [14]. However, further application of this reaction was hindered by the low yields and the lack of reaction details. During the same period, the first metal-dinitrogen (N_2_-M) complex [Ru(NH_3_)_5_(N_2_)]^2+^ was reported in 1965 [15]. After that, thousands of N_2_-M complexes have been documented [16]. The reactivity exploration reveals that the functionalization of the N_2_ ligands can also be fulfilled for some N_2_-M complexes [17]. Making N−C bonds from the reactions of transition metal N_2_ complexes with carbon-based reagents has received much attention in recent decades, although the catalysis system has not been realized [18,19].

**Figure 1.**
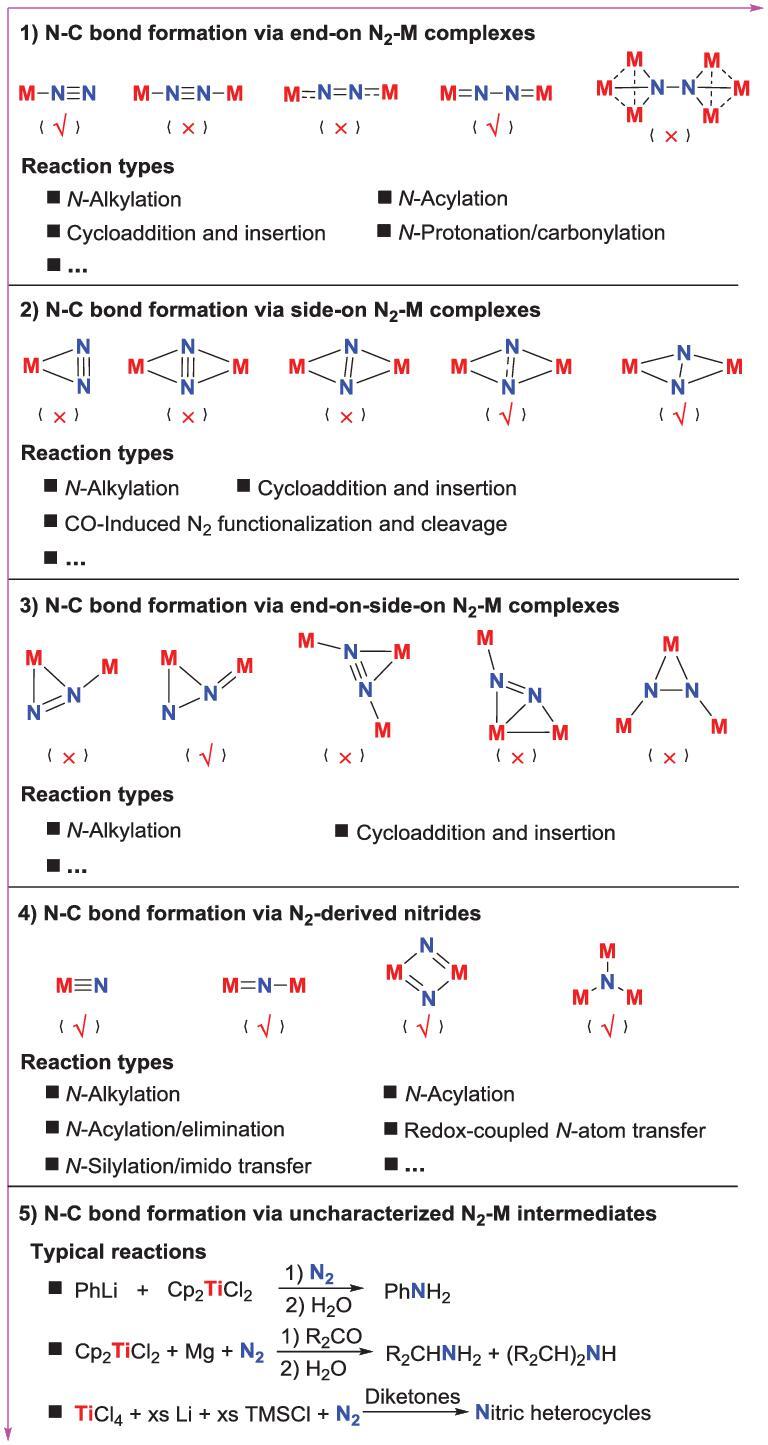
The classification of this review. The N−C bond formation is reported (√) or not reported (×).

This review will focus on the previous works regarding the transformation of N_2_ into organic compounds. In almost all of these works, the N−C bond formation steps are fulfilled upon the well-defined N_2_-M complexes with diversified coordination modes. This review is organized by the coordination modes of the N_2_-M complexes (to clarify, the N_2_-derived metal nitrides are also considered as a coordination mode of N_2_-M complexes) that are involved in the N−C bond formation steps, and each part is arranged in terms of the type of reactions between N_2_-M complexes and carbon-based substrates. The earlier works about one-pot synthesis of organic compounds from N_2_ via ill-defined intermediates are also introduced briefly in this review (Fig. 1).

## N−C BOND FORMATION VIA END-ON N_2_-M COMPLEXES

End-on bond is the most prevalent bonding mode for N_2_-M complexes and the N_2_-M complexes with this binding mode have been known to assemble N−C bond for a long time. Main works were achieved via the reaction of end-on terminal N_2_-M complexes with alkyl or acyl halides and their analogues. N−C bond formations from the cycloaddition and insertion reactions of end-on-bridged N_2_-M complexes with imido-like N_2_ ligands have also been reported.

### *N*-alkylation

#### *N*-alkylation by electrophiles

**Scheme 1.**
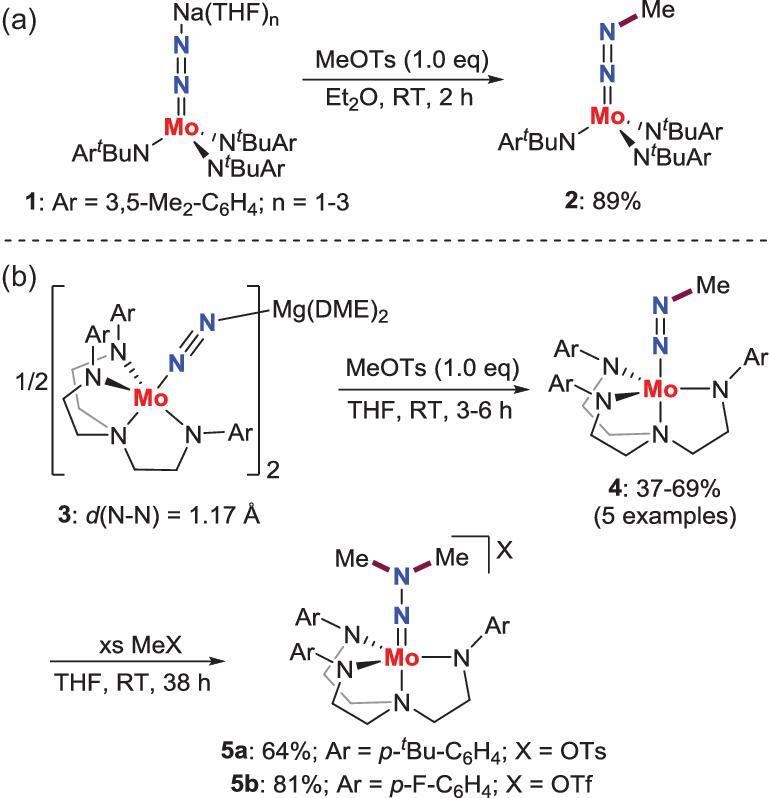
*N*-alkylation of end-on terminal N_2_-Mo complexes by electrophiles. (a) *N*-methylation of N_2_-Mo complex by MeOTs to afford methyldiazenido complex. (b) *N*-methylation of N_2_-Mo complexes by MeOTs and MeOTf to afford methyldiazenido and *N*,*N*-dimethylhydrazido complexes.

The strong electrophilic alkyl triflates and their analogues are often employed to functionalize the end-on N_2_-M complexes because the N_2_ ligands in these complexes feature a nucleophilic character by electron donation from the electron-rich metal centers. Peters *et al.* [20] and Greco and Schrock [21] reported that the methylation reaction occurs when the anionic end-on N_2_-Mo complexes **1** and **3** are treated with methyl tosylate (MeOTs) to provide methyldiazenido complexes **2** and **4** (Scheme 1a and b). Additionally, **4** could further undergo N−C bond formation to furnish *N*,*N*-dimethylhydrazido complexes **5** by reaction with excess methyl triflate (MeOTf) or MeOTs (Scheme 1b).

**Scheme 2.**
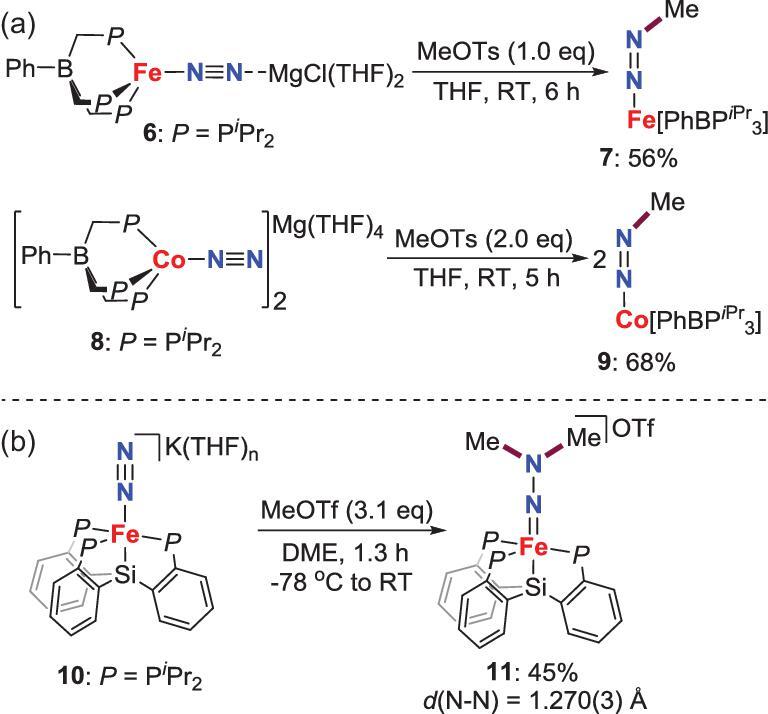
*N*-alkylation of end-on terminal N_2_-Fe, Co complexes by MeOTf or MeOTs. (a) *N*-methylation of N_2_-Fe, Co complexes by MeOTs to afford methyldiazenido complexes. (b) *N*-methylation of N_2_-Fe complexes by MeOTf to afford *N*,*N*-dimethylhydrazido complex.

Although many late-transition metal complexes with end-on N_2_ ligands have been documented, reports on their reactivity toward electrophiles to make N−C bond are very rare. Peters *et al.* described that the anionic end-on terminal N_2_ complexes of Fe **6** and Co **8** react with MeOTs to give *N*-methylation species **7** and **9** (Scheme 2a) [22]. In 2016, the same group found that the modified N_2_-Fe complex **10** bearing monoanionic tetradentate trisphosphinosilyl ligand can also be alkylated to afford *N,N*-dimethylated product **11** (Scheme 2b) [23].

#### *N*-alkylation by *in situ* formed radicals

**Scheme 3.**
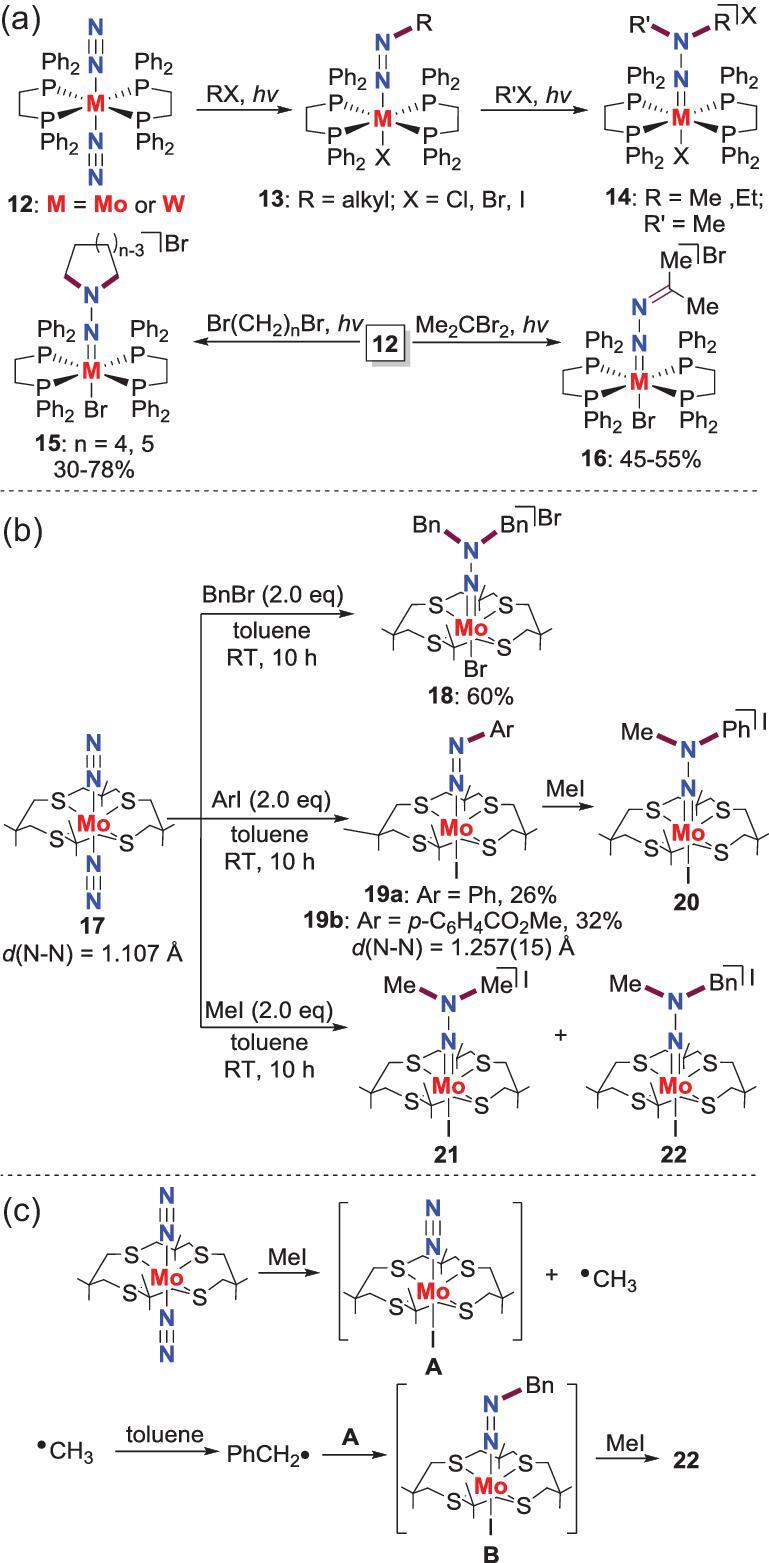
*N*-alkylation of end-on terminal N_2_-Mo, W complexes by *in situ* formed radicals. (a) *N*-alkylation of N_2_-Mo, W complexes supported by diphosphine ligands. (b) *N*-alkylation of N_2_-Mo complex supported by tetra-thioether ligand. (c) A plausible mechanism for the generation of **22**.

There are only a few examples of N−C bond formation at N_2_-M complexes by radicals. One example is the reactions of terminal end-on N_2_-Mo, W complexes **12** with alkyl halides, driven by light (*vide infra*). Mechanism investigation reveals that the radicals in these reactions are generated *in situ* by the homolysis of the alkyl halides within the coordination sphere. The attacking of these alkyl radicals at the N_2_ ligands provides **13**. Furthermore, dialkylhydrazido complexes **14**, **15** and **16** can also be obtained via alkylation of **13** or one-pot dialkylation of **12** (Scheme 3a) [24,25]. It is noteworthy that if the diphosphine ligands in **12** are replaced by the monophosphine ligands, the corresponding N_2_-Mo, W complexes fail to react with alkyl halides to assemble N−C bond. Another example that involves the radical mechanism is the *N*-functionalization of the terminal end-on N_2_-Mo complex **17**, which possesses higher reactivity than **12** (Scheme 3b) [26]. For instance, the treatment of **17** with BnBr or aryl iodide gives the *N*,*N*-dibenzylation product **18** or *N*-arylation complex **19,** the latter of which can also be converted to the organo-hydrazido species **20** by further reaction with MeI. More intriguingly, when **17** is treated with MeI in toluene, the prospective product **21** is formed together with isolation of an unexpected product **22**. A plausible mechanism is raised for the generation of **22** (Scheme 3c). The initial reaction between **17** and MeI results in iodine atom abstracting to afford intermediate **A** and the methyl radical, which would abstract an *H*-atom from toluene to yield benzyl radical. The latter reaction between **A** and benzyl radical gives the *N*-benzylation intermediate **B**, which can further react with MeI to afford the final product. The formation of **22** confirms the radical process of these *N*-alkylation reactions again.

#### *N*-alkylation by nucleophiles

**Scheme 4.**
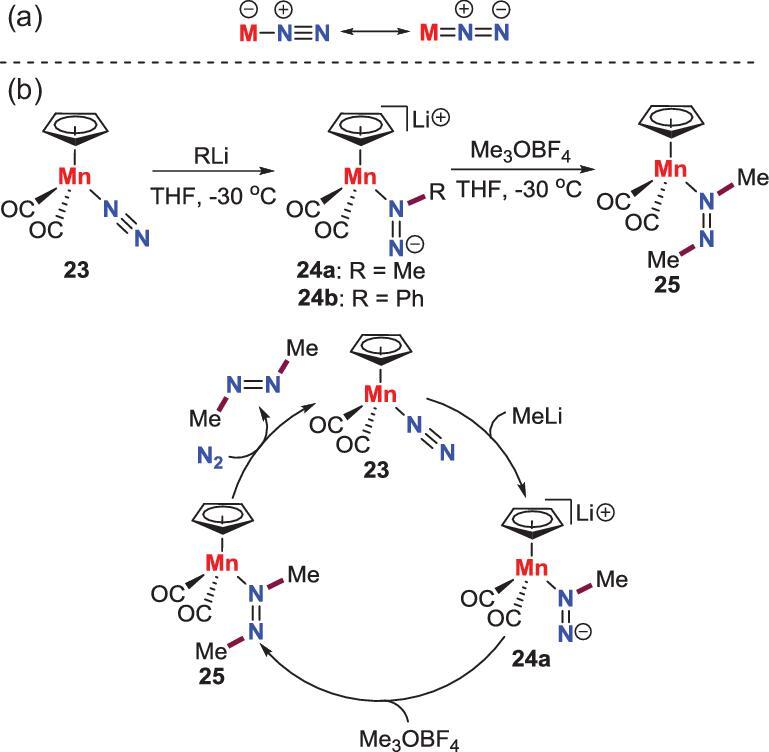
Manganese-promoted direct conversion of N_2_ into azomethane via the reaction between nucleophiles and N_2_-Mn complex. (a) Simple Lewis formulas for end-on terminal N_2_-M complexes. (b) A synthetic cycle for synthesis of azo-compound from N_2_.

For end-on terminal N_2_-M complexes, simple Lewis formulas could be used to depict their structures. As shown in Scheme 4a, the *N* atom adjacent to the metal atom (N_α_) features positive charge and could be attacked by nucleophiles in theory. Surprisingly, there is only one example of this reactivity for N_2_-M complexes [27,28]. Sellman *et al*. found that an end-on terminal N_2_-Mn complex **23** reacts with methyl or phenyl lithium reagent at low temperature to give the *N*_α_-functionalized products **24**, which could subsequently react with Meerwein reagent Me_3_OBF_4_ upon *N*_β_ atom to afford **25**. This azomethane complex would ultimately liberate free azomethane by pressuring with 100 bar of N_2_ along with reforming N_2_-Mn complex **23**. Thus, a synthetic cycle was raised for synthesis of azo-compound from N_2_.

### *N*-acylation

**Scheme 5.**
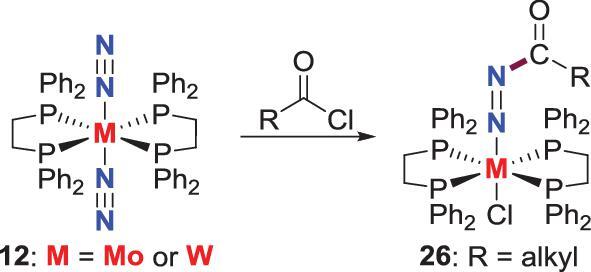
*N*-acylation of end-on terminal N_2_-Mo, N_2_-W complexes.

Besides alkyl halides, acyl chlorides are also used to functionalize end-on N_2_-M complexes. Chatt *et al.* found that the N_2_-Mo, W complexes **12** supported by bidentate phosphines ligands react with acyl chloride to afford acyldiazenido complexes (Scheme 5) [29,30]. These *N*-acylation reactions possibly proceed through nucleophilic attacking of the N_2_ ligands on the acyl carbons.

### Cycloaddition and insertion

**Scheme 6.**
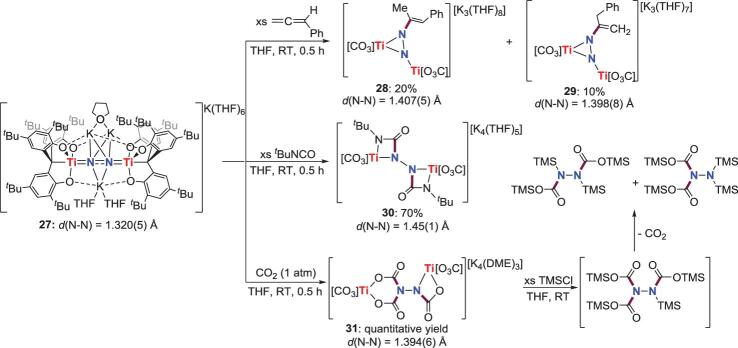
N−C bond formation from cycloaddition of end-on-bridged N_2_-Ti complex with phenylallene, *^t^*BuNCO and CO_2_.

For some end-on-bridged N_2_-M complexes with strongly activated N_2_ ligands, the imido-like structures make them able to undergo cycloaddition or insertion reactions with carbon-based unsaturated substrates to assemble N−C bond. In 2017, the reaction of an end-on bridging binuclear N_2_-Ti complex **27** with phenylallene, *tert*-butyl isocyanate (*^t^*BuNCO) and CO_2_ was investigated by  Kawaguchi *et al*., to provide N−C bond formation products (Scheme 6) [31]. Treatment of **27** with an excess of phenylallene results in the formation of dititanium hydrazido complexes **28** and **29** as a mixture of isomers. The formation of **28** and **29** can be rationalized in terms of an initial [2+2] cycloaddition of phenylallene with Ti = N bond in **27** to give the 4-membered titanacycle intermediates (two isomers), and the subsequent protonolysis of the Ti−C bonds in these intermediates to give the final products. Further studies indicate that the proton source in this reaction could be a second equivalent of phenylallene, the ancillary ligands, or even adventitious impurities present in the reaction mixture. The reaction of **27** with *^t^*BuNCO also proceeds through a formal [2+2] cycloaddition reaction to afford **30**. However, when **27** is introduced with an atm of CO_2_, the insertion of three molecules of CO_2_ into Ti = N bonds in **27** is achieved to furnish **31**. By adding an excess amount of TMSCl, **31** could be converted to organic compound N_2_(TMS)(CO_2_TMS)_3_, which is unstable under the reaction condition and decomposes to two hydrazine derivatives [TMS(CO_2_TMS)N]_2_ and (TMS)_2_NN(CO_2_TMS)_2_ via decarboxylation.

**Scheme 7.**
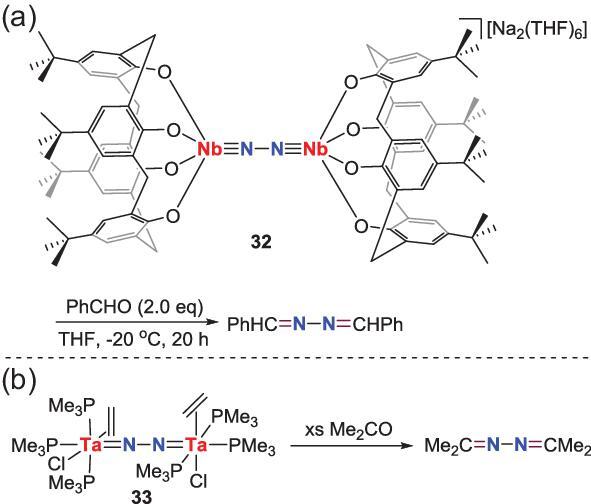
N−C bond formation from the reactions of end-on-bridged N_2_-Nb, Ta complexes with aldehyde or acetone. (a) The reaction of N_2_-Nb complex with benzaldehyde. (b) The reaction of N_2_-Ta complex with acetone.

The cycloaddition reactions between group 5 end-on N_2_-M complexes and carbon-based unsaturated bonds have also been observed. For example, N_2_-Nb complex **32** and N_2_-Ta complex **33** with diimido bridging N_2_ ligands are known to react with aldehyde and acetone to afford the corresponding ketazines (Scheme 7) [32,33].

### *N*-protonation/carbonylation

**Scheme 8.**
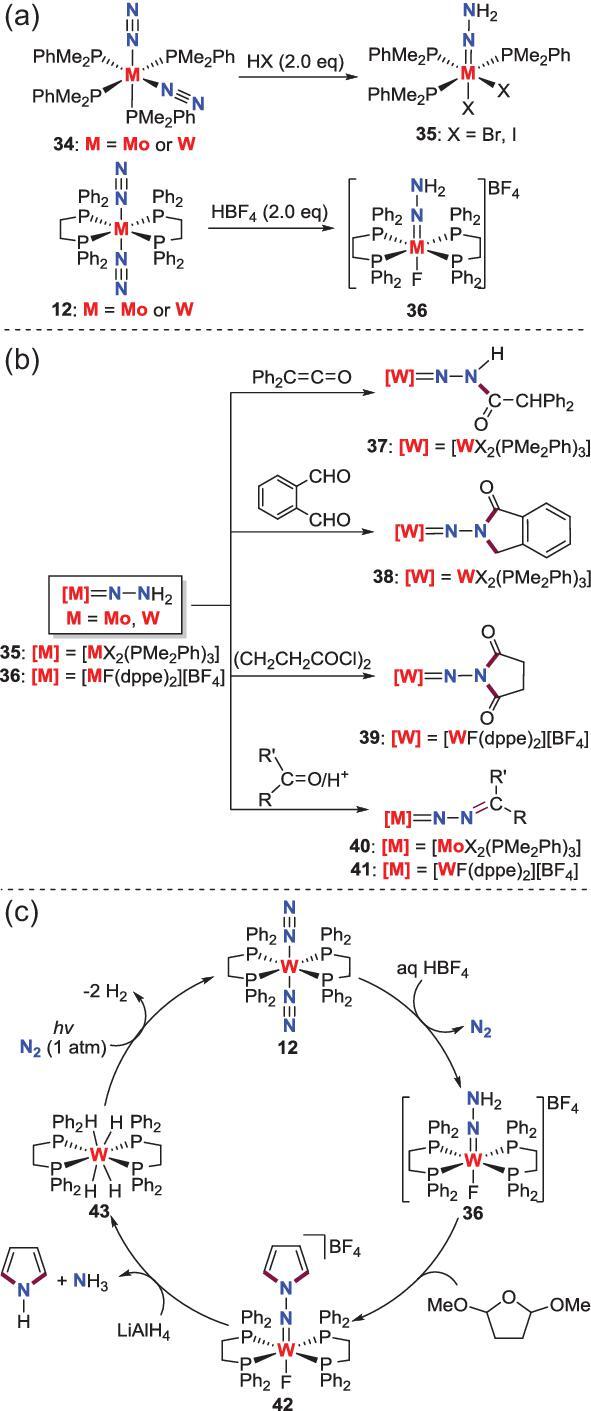
N−C bond formation from the reactions of hydrazido Mo, W complexes with carbon-based reagents. (a) The reaction of N_2_-Mo, W complexes with HX (X = Cl, Br and I) or HBF_4_ to afford the hydrazido complexes. (b) Carbonylation of hydrazido complexes **35** and **36** to assemble N−C bond. (c) A synthetic cycle for synthesis of 1H-pyrrole from N_2_.

An alternative route for making N−C bond is the treatment of carbon-based substrates with the *N*-hydrogenated complexes derived from N_2_ because in some cases *N*-hydrogenation are more accessible than *N*-alkylation for end-on N_2_-M complexes. Seminal works about these transformations were finished by Hidai and others [25,34]. They reported that the N_2_-Mo, W complexes **34** and **12** supported by monophosphine or diphosphine ligand react with HX (X = Cl, Br and I) or HBF_4_ to afford the hydrazido complexes **35** and **36**, which can act as the versatile precursors to construct N−C bond [25,35] (Scheme 8a). For instance, **35** could react with diphenylketene and phthalaldehyde to provide **37** and **38**, while the reaction between **36** and succinyl chloride gives rise to **39**. More intriguingly, these hydrazido complexes **35** and **36** are also reported to undergo a condensation reaction with ketones and aldehydes in the presence of catalytic amounts of acid to afford all kinds of diazoalkane complexes **40** and **41** (Scheme 8b). The liberation of the *N*-containing organic compounds from these *N*-functionalized complexes has also been explored [36]. For example, when the cyclic hydrazido complex **42**, produced from the reaction of **36** and the cyclic acetal of succinaldehyde, is treated with LiAlH_4_, the reductive destruction of **42** is observed to release 1H-pyrrole accompanied by the generation of the tetrahydride complex **43**. Furthermore, this tetrahydride tungsten complex could be converted to the initial N_2_-W complex **12** under photolytic conditions to achieve a cycle (Scheme 8c) [37].

### Involvement of photochemistry

Photochemistry is an emerging approach for the transfer of N_2_. The earliest observation of photo-catalyzed N−C bond formation of N_2_-M complexes is of the reactions between end-on terminal N_2_-M complexes **12** and alkyl halides (Scheme 3a) [25]. In the case of N_2_-W complex, visible light or a tungsten-lamp is often necessary for these *N*-alkylation reactions. However, for the N_2_-Mo complex, it could react with alkyl bromide slowly in the dark. It is also reported that the N_2_ ligands in **12** are not evolved in the absence of the alkyl halides since irradiation of the N_2_-M complexes without organic halide caused no change. These results indicate the possibility of photo engaging in the assistance of alkyl radicals formation in these reactions [24].

### Involvement of electrochemistry

**Scheme 9.**
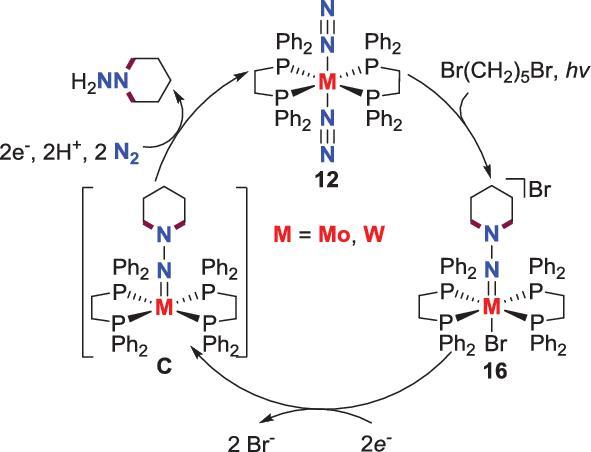
An electrochemical cycle for synthesis of piperidine direct from N_2_ via end-on terminal N_2_-Mo, W complexes.

Besides photochemistry, electrochemistry is another versatile method in the N_2_ conversion process. Although the direct involvement of electrochemistry in the N−C bond formation step has not been discovered, the electrochemical reduction of the *N*-alkylated complexes to release the final organic products has been developed. For example, the organohydrazido complexes **16**, which is synthesized from the reaction of N_2_-M complexes **12** with 1,5-dibromopentane, undergoes electrochemical reduction at a Pt electrode in tetrahydrofuran (THF) under N_2_ by using [NBu_4_][BF_4_] as the electrolyte to liberate piperidine accompanied by the regeneration of N_2_-M complexes **12**. According to the control experiment under the atmosphere of Ar or CO, a M(II) hydrazido intermediate is proposed in this piperidine releasing process. Based on these results, an electrochemical cycle to synthesize dialkylhydrazine from N_2_ was reported by Leigh *et al*. (Scheme 9) [38].

## N−C BOND FORMATION VIA SIDE-ON N_2_-M COMPLEXES

The side-on bonding modes are often observed at group 3 and group 4 transition metal N_2_-M complexes. The N_2_ ligands in these side-on N_2_-M complexes can be divided into neutral N_2_, (N_2_)^2−^, (N_2_)^3−^ and (N_2_)^4−^, and the N−C bond formation usually takes place at side-on-bridged binuclear N_2_-M complexes with (N_2_)^4−^ moiety. Very recently, the *N*-functionalization of a (N_2_)^3−^-Sc complex was also fulfilled.

### *N*-alkylation

**Scheme 10.**
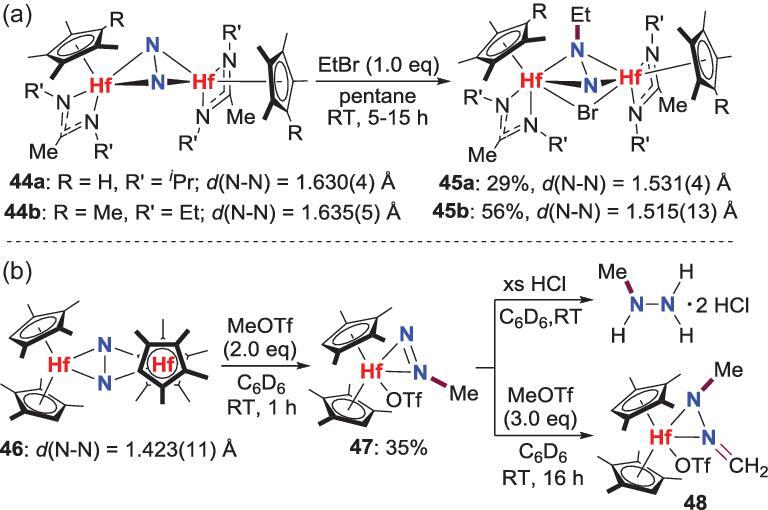
*N*-alkylation of side-on-bridged N_2_-Hf complexes by EtBr or MeOTf. (a) *N*-ethylation of N_2_-Hf complex by EtBr. (b) N-methylation of N_2_-Hf complex by MeOTf.

There are two reports about the reaction of group 4 side-on N_2_-M complexes with alkyl halides or their analogues to make N−C bond. One example was reported by Hirotsu *et al*. in 2007, in which the side-on-bridged N_2_-Hf complexes **44** with extremely activated (N_2_)^4−^ ligands can react with ethyl bromide (EtBr) to provide the *N*-ethylated products **45** (Scheme 10a) [39]. Controlled experiments indicate that this reaction is remarkably sensitive to the steric effects of the ancillary ligands. For example, when the R’ group in **44b** is changed from Et to *^i^*Pr, the corresponding *N*-ethylation product could not be obtained. Besides, **45a** and **45b** fail to undergo further *N*-ethylation, even in the presence of excess EtBr. The other work is reported by the reaction of methyl triflate (MeOTf) with a hafnocene complex **46** that also bears side-on bridging (N_2_)^4−^ ligand (Scheme 10b) [40]. This reaction offers a mixture of products and one of them is the N_2_ ligand mono-methylated product **47**, which could be converted to the final organic compound *N*-methylhydrazine by treating with excess HCl. Besides, an unprecedented triflato hafnocene hydrazonato complex **48** is generated via a second N−C bond formation when additional MeOTf is added to **47**.

**Scheme 11.**
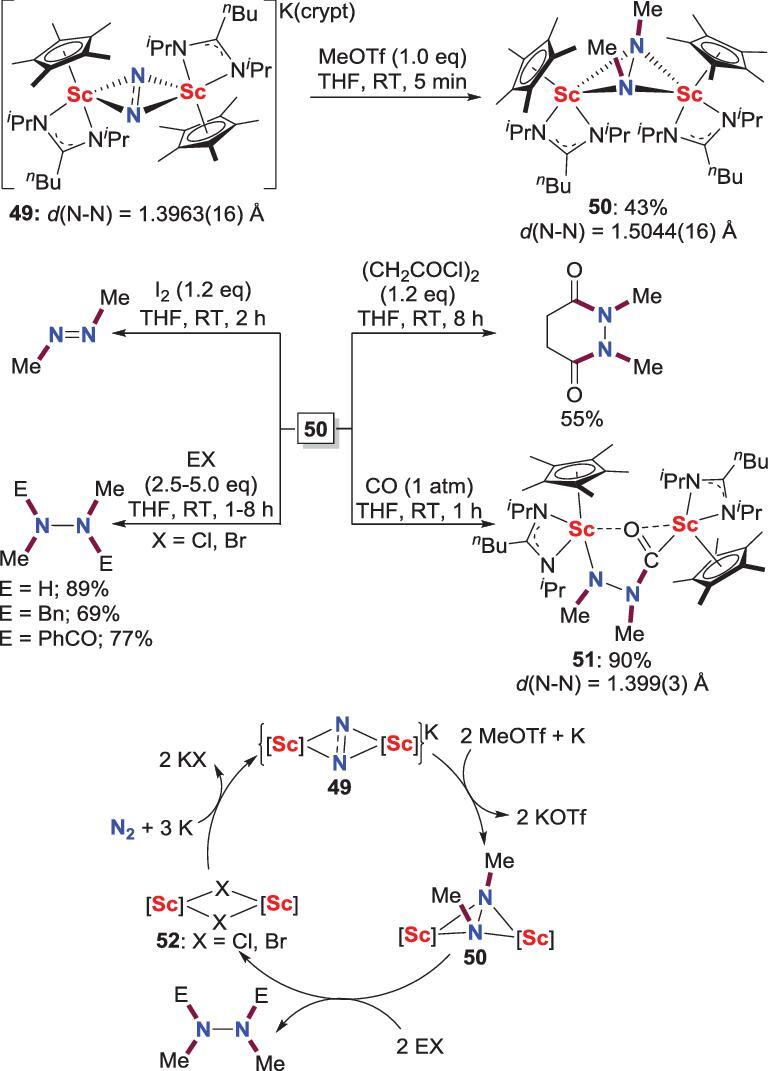
Scandium-promoted direct conversion of N_2_ into hydrazine derivatives via the reaction between MeOTf and N_2_-Sc complex.

Compared with the group 4 transition metals, rare-earth metal promoted direct conversion of N_2_ into organic compounds attracts less attention. The only example of this topic was reported by Xi, Zhang *et al.* in 2019 (Scheme 11) [41]. Treatment of the (N_2_)^3−^-bridged discandium complex **49** with MeOTf leads to the formation of *N*,*N*^′^-dimethylation discandium complex **50** in 43% yield. The yield of **50** can be improved via the treatment of **49** with MeOTf and potassium several times. Transformation of the (N_2_Me_2_)^2−^ ligand into organic compounds could be accomplished by treatment of **50** with I_2_, HCl, BnBr and acyl chloride to afford azomethane, 1,2-dimethylhydrazine and a series of tetra-substituted hydrazine derivatives, concomitant with the regeneration of the precursors of the N_2_-Sc complexes. Hence, a three-step synthetic cycle for scandium-mediated direct conversion of N_2_ and carbon-based electrophiles to multi-substituted hydrazine derivatives could be realized. The insertion of a CO molecule into the Sc−N bond of **50** with further N−C bond formation is also observed to provide **51**.

### Cycloaddition and insertion

**Scheme 12.**
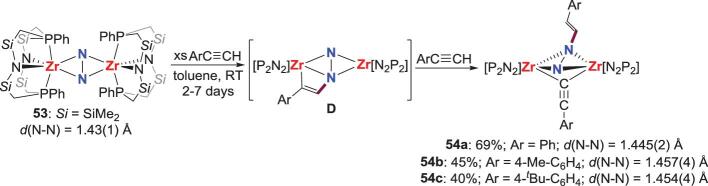
N−C bond formation of side-on N_2_-Zr complex by reaction with alkynes.

The group 4 side-on bridging N_2_-M complexes are known to undergo cycloaddition and insertion reactions with carbon-based reagents that contain C = X (X = N, O) bonds or C≡C bond, such as carbon dioxide (CO_2_), isocyanates (RNCO) or alkynes, owing to their imido-like reactivity. Compared with *N*-alkylation and acylation reactions, these cycloaddition and insertion reactions are more atom-efficient for N_2_ functionalization, because the formation of transition metal halides and inorganic salts as the by-products is avoided in these reactions. The earliest study of the cycloaddition reactions between group 4 N_2_-M complexes and unsaturated bond to assemble N−C bond was finished by Fryzuk *et al.* via the reaction of side-on bridging N_2_-Zr complex **53** with arylacetylene (RC≡CH; R = Ph, 4-Me-C_6_H_4_ and 4-*^t^*Bu-C_6_H_4_) (Scheme 12) [42]. The *N*-functionalization products **54** may result from a sequence of two successive steps: cycloaddition of alkyne across a Zr−N bond in **53** leading to the zircona-aza-cyclobutene intermediate **D**, which subsequently encounters Zr−C bond cleavage by protonation with another molecular terminal alkyne to yield **54**.

**Scheme 13.**
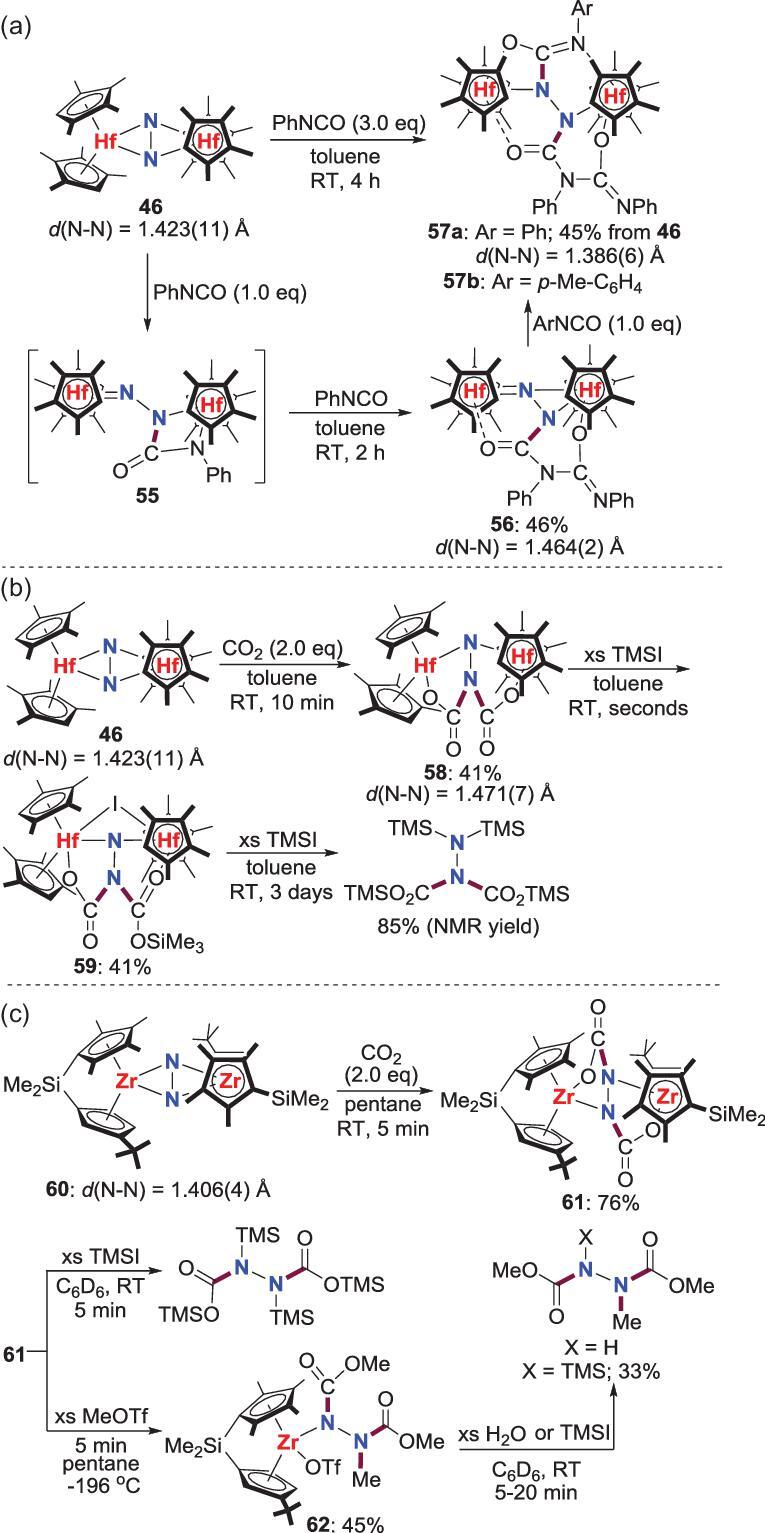
N−C bond formation from the reactions of the side-on N_2_-Zr, Hf complexes with isocyantes and CO_2_. (a) The reaction of N_2_-Hf complex with PhNCO. (b) The reaction of N_2_-Hf complex with CO_2_. (c) The reaction of N_2_-Zr complex with CO_2_.

By elegant modulation of the substitutions on the multi-substituted Cp ligands, Chirik *et al*. accomplished a series of reactions of dinuclear N_2_-Zr, Hf complexes with isocyanates or CO_2_ to assemble N−C bond. N_2_-Hf complex **46** bearing tetramethylcyclopentadienyl (Cp^4Me^) ligand is reported to react with PhNCO to provide the initial product **56** via a possible intermediate **55**. In the solution, **56** also reacts quickly with another molecule of ArNCO (Ar = Ph and *p*-MeC_6_H_4_) to afford **57**, which could also be prepared directly from **46** (Scheme 13a) [43]. Besides, further studies indicate that another *N*-functionalization product **58**, in which the same nitrogen atom is di-carboxylated, would be formed predominately when CO_2_ is bubbled into a solution of **46** (Scheme 13b) [44]. Subsequent reaction of **58** with TMSI gives rise to the generation of **59**, which is known to liberate the corresponding hydrazine derivative (TMS)_2_NN(CO_2_TMS)_2_ by further reacting with excess TMSI. Unfortunately, the similar *N*-functionalization reactions of PhNCO and CO_2_ with zirconium congener of **46** are unsuccessful, which is believed to be caused by the deleterious ligand-induced side-on, end-on isomerization of the (N_2_)^4−^ ligand. Hence, a [Me_2_Si]-bridged *ansa*-zirconocenes N_2_ complex **60** with higher energy barrier for the side-on, end-on isomerization was designed and prepared to investigate the reactivity toward CO_2_ [45]. The treatment of **60** with CO_2_ leads to the immediate generation of **61**, where N_2_-functionalization takes place at each *N*-atom. Organic compound *N*,*N*^′^-dicarboxylated hydrazine can be released from **61** by reacting with TMSI. Furthermore, a second N−C bond formation occurs when **61** is treated with MeOTf to provide **62**, which is known to liberate region-specific hydrazine X(COOMe)NN(COOMe)Me (X = H, TMS) by further reacting with H_2_O or TMSI (Scheme 13c). These results indicate that small modifications of the ligands will change the reactivity of the N_2_-M complexes dramatically.

### CO-induced N_2_ functionalization and cleavage

Being isoelectronic with N_2_, CO is an abundant and cheap diatomic molecule with BDE of 1 079 kJ/mol. Hence, the transformation of CO and N_2_ into N−C bond is a challenge but a significant process. Until now, only two systems of CO-induced N_2_ ligand scission and functionalization at N_2_-M complexes have been developed, in which all of the N_2_ ligands adopt side-on bridging coordination mode.

**Scheme 14.**
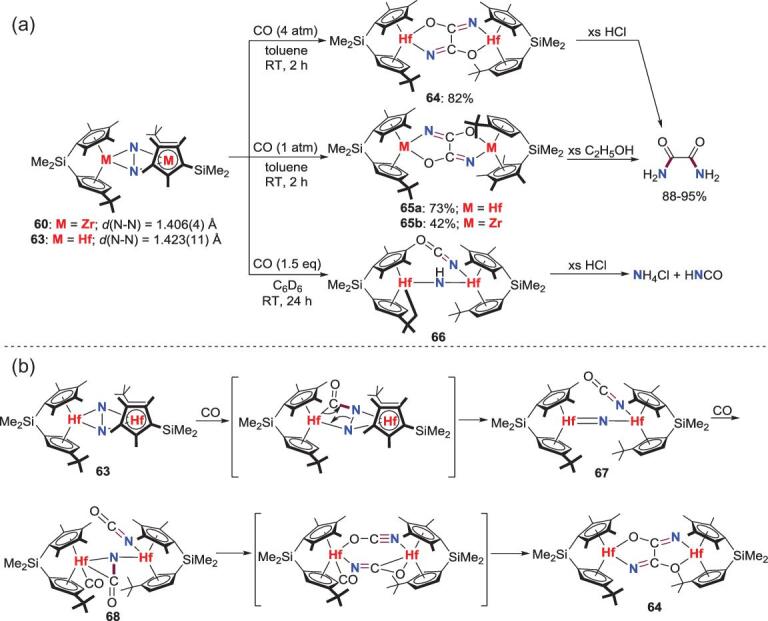
CO-induced N_2_ scission and functionalization at side-on N_2_-Zr and Hf complexes. (a) The reaction of N_2-_Zr, Hf complexes and CO. (b) A plausible mechanism for this CO-induced N_2_ scission and functionalization reaction.

Following their earlier work on N−C bond formation from N_2_-Zr and Hf complexes, Chirik *et al*. reported in 2010 the first example that treatment of the *ansa*-zirconocene and hafnocene N_2_ complexes **60** and **63** with 4 atm or 1 atm of CO leads to the generation of the dinuclear oxamidide complexes **64** and **65** as two isomers [46,47]. Besides, when **63** is treated with less CO (1.5 equiv), a new product of imido-bridged dihafnium complex **66** could be isolated, in which the *H*-atom on the bridging imido is derived from the cyclometallation of the *^t^*Bu group (Scheme 14a). Protonolysis enables these products to release the corresponding *N*-containing organic compounds of free oxamide and isocyanic acid. Density functional theory (DFT) calculations [48] and experimental results [49] reveal that the formation of **64** and **65** is assumed to be initiated by CO insertion into an Hf−N bond and followed by the retro [2+2] cycloaddition to provide the presumptive *μ*-nitride species **67**. The coordination and insertion of CO to the *μ*-nitrido intermediate **67** results in the formation of **68**, which was characterized by multinuclear nuclear magnetic resonance (NMR) spectroscopy at low temperature. **68** undergoes C−C bond formation via coupling of the terminal and bridging isocyanate units along with the loss of the terminal carbonyl ligand to give the final products (Scheme 14b).

**Scheme 15.**
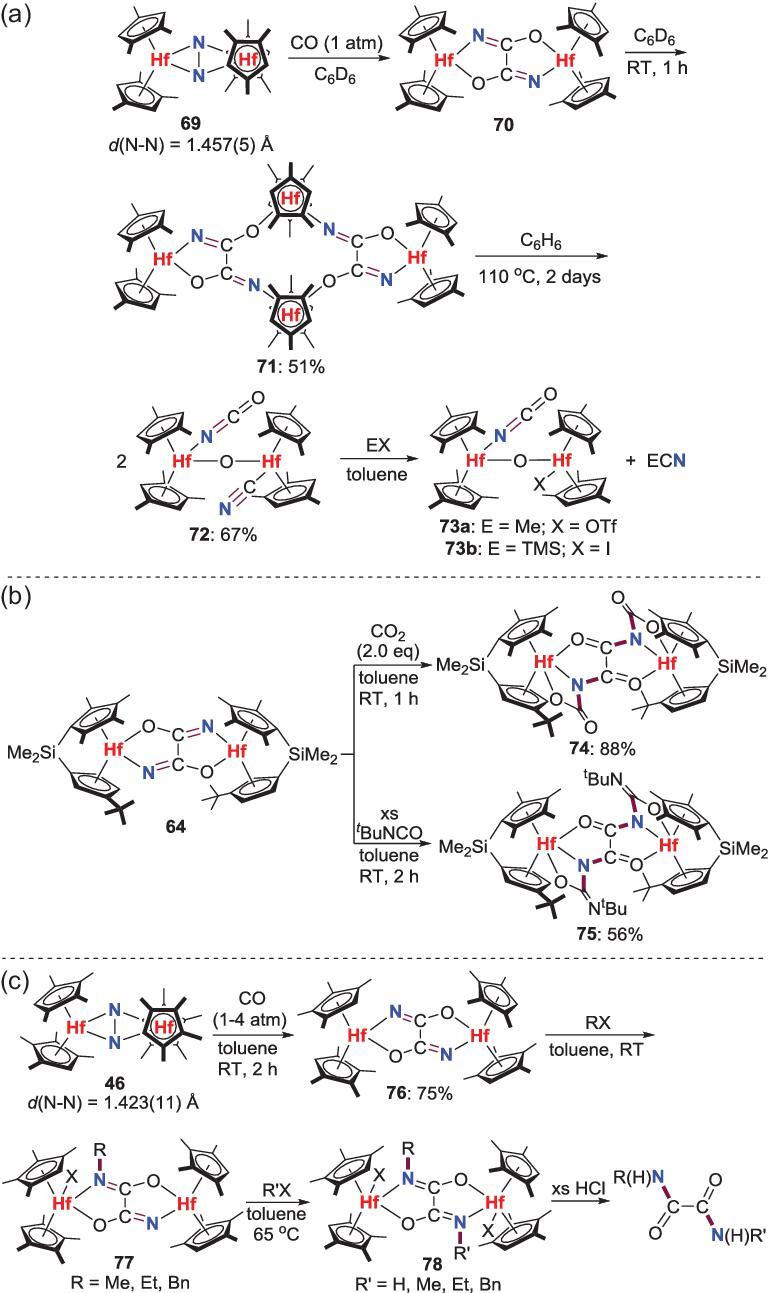
N−C bond formation of CO- and N_2_-derived oxamidide complexes. (a) Thermolysis of oxamidide complex **71**. (b) The reaction of oxamidide complex **64** with CO_2_ and *^t^*BuNCO. (c) *N*-alkylation of the oxamidide complex **76** to afford *N*,*N*^′^-dialkyloxamides.

More studies indicate that these CO-induced N_2_ cleavage and functionalization reactions are also compatible with other zirconocene and hafnocene N_2_ complexes. Therefore, a tetrametallic hafnocene oxamidide complex **71** could be obtained via a dimeric hafnium intermediate **70** when the N_2_-Hf complex **69** is treated with CO [50]. The transformations of these oxamidide complexes were also elaborated. Thermolysis of **71** at 110°C provides a *μ*-oxo hafnocene complex **72** with both terminal cyanide and isocyanate ligands that undergoes preferential group transfer of the cyanide unit to liberate organonitriles of TMSCN or MeCN along with the generation of **73** by reacting with TMSI or MeOTf (Scheme 15a) [50]. Oxamidide complex **64** reacts with CO_2_ and *^t^*BuNCO to give the formal [2+2] cycloaddition products **74** and **75** [51] (Scheme 15b). Additionally, various free *N*,*N*^′^-dialkyloxamide could be formed via stepwise *N*-alkylation of the oxamidide complex **76** and following protonolysis with HCl or ethanol (Scheme 15c).

**Scheme 16.**
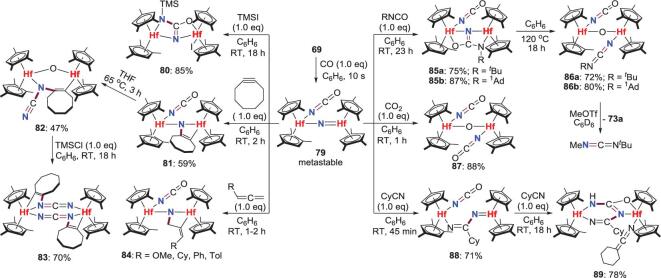
N−C bond formation of CO- and N_2_-derived Hf-nitride complexes.

The characterization and reactivity studies of the *μ*-nitride intermediates were also developed (Scheme 16). Rapid bubbling of CO into N_2_-Hf complex **69** at a low temperature produces a metastable dihafnocene nitride complex **79**, which is characterized by IR and multinuclear NMR spectroscopy. This base-free *μ*-nitride can react with various substrates [52–55]. For instance, the treatment of **79** with TMSI affords silylureate complex **80**. This reaction is involved in the initial iodide ion abstraction to give a transient silyl cation and a formally anionic bridging nitride intermediate, whose nucleophilicity is increased by weakening the Hf−N multiple bonding. Hence, this intermediate could undergo nucleophilic attacking of the nitride group to the terminal isocyanate moiety to form the ureate core, which is then trapped by the silyl cation to yield **80**. Besides, when **79** is treated with cyclooctyne, monosubstituted allenes and isocyanates, the formal [2+2] cycloaddition reactions occur to afford **81**, **84** and **85**, respectively. The alkyne and isocyanates products are kinetically unstable at elevated temperature and engage in additional N−C bond formations to give **82** and **86**. Complex **82** could be converted to a binuclear complex **83** with two bridging carbodiimidyl ligands by reacting with TMSCl. The *μ*-oxo complex **86** can liberate *N*-containing organic compound of carbodiimide along with the generation of dihafnium oxo complex **73** by reacting with MeOTf. In contrast, exposure of the nitride complex **79** to another heterocummulene of CO_2_ provides *μ*-oxo bis(isocyanate) complex **87**, resulting from deoxygenation of CO_2_ accompanied by N−C bond formation. Furthermore, the Hf−nitride bond in **79** also engages in the insertion of cyclohexylnitrile (CyCN) to provide **88**, which can continue reacting with another molecule of CyCN to afford bridging ureate-type complex **89** via additional N−C bond formation (Scheme 16).

**Scheme 17.**
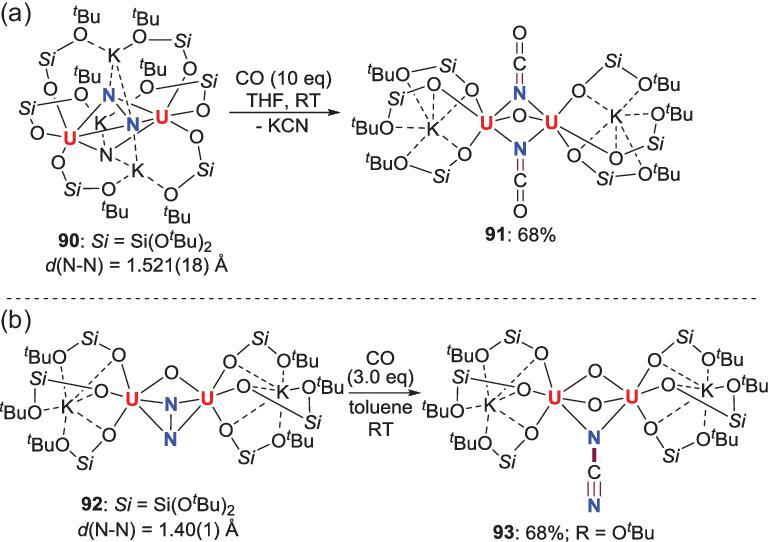
CO-induced N_2_ scission and functionalization at side-on N_2_-U complexes. (a) The reaction between CO and N_2_-U complex **90** with *μ*-nitride ligand. (b) The reaction between CO and N_2_-U complex **92** with *μ*-oxo ligand.

The other system of CO-induced N_2_ scission and functionalization was discovered by Mazzanti *et al*. using uranium complexes. A side-on-bridged binuclear N_2_-U complex **90** with *μ*-nitride ligands reacts with CO to provide the oxo/cyanate diuranium complex **91** accompanied by releasing of potassium cyanate (KCN), which is formed from the reaction of nitride unit with CO [56] (Scheme 17a). To understand the role of the bridging nitride in these transformations, a similar N_2_-U complex **92** with bridged *μ*-oxo ligand was synthesized and its reactivity toward CO was also investigated [57]. The reaction between **92** and CO immediately results in the generation of cyanamido bridged complex **93** with retaining the *μ*-oxo moiety via both cleavages of N−N single bond and C≡O triple bond (Scheme 17b). DFT calculation indicates that the different reactivity of **90** and **92** is attributed to the different bonding nature of the N_2_ ligands, in which the *μ*-nitride is involved in the binding and resultant activation of N_2_, but the *μ*-oxo is not.

## N−C BOND FORMATION VIA SIDE-ON-END-ON N_2_-M COMPLEXES

The side-on-end-on bound mode is much less common relative to the aforementioned two coordination modes in N_2_-M complexes. All of the work regarding the making of N−C bond from N_2_-M complex with this bonding mode were finished by Fryzuk *et al*. by employing a binuclear N_2_-Ta complex.

### *N*-alkylation

**Scheme 18.**
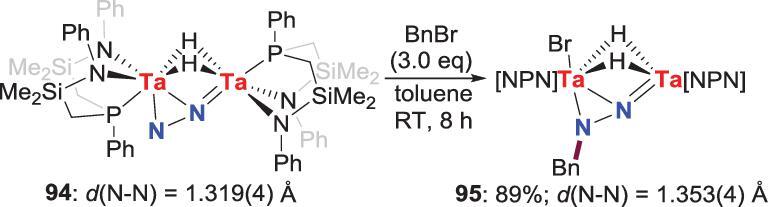
*N*-alkylation of side-on-end-on N_2_-Ta complex by BnBr.

In 2001, the *N*-alkylation of the side-on-end-on bridging binuclear N_2_-Ta complex **94** was developed to afford *N*-benzylation product **95** in high yield by reaction with benzyl bromide (BnBr) (Scheme 18) [58]. This reaction was similar to the *N*-alkylation reaction of the side-on N_2_-Zr complexes **44** (Scheme 10a).

### Cycloaddition and insertion

**Scheme 19.**
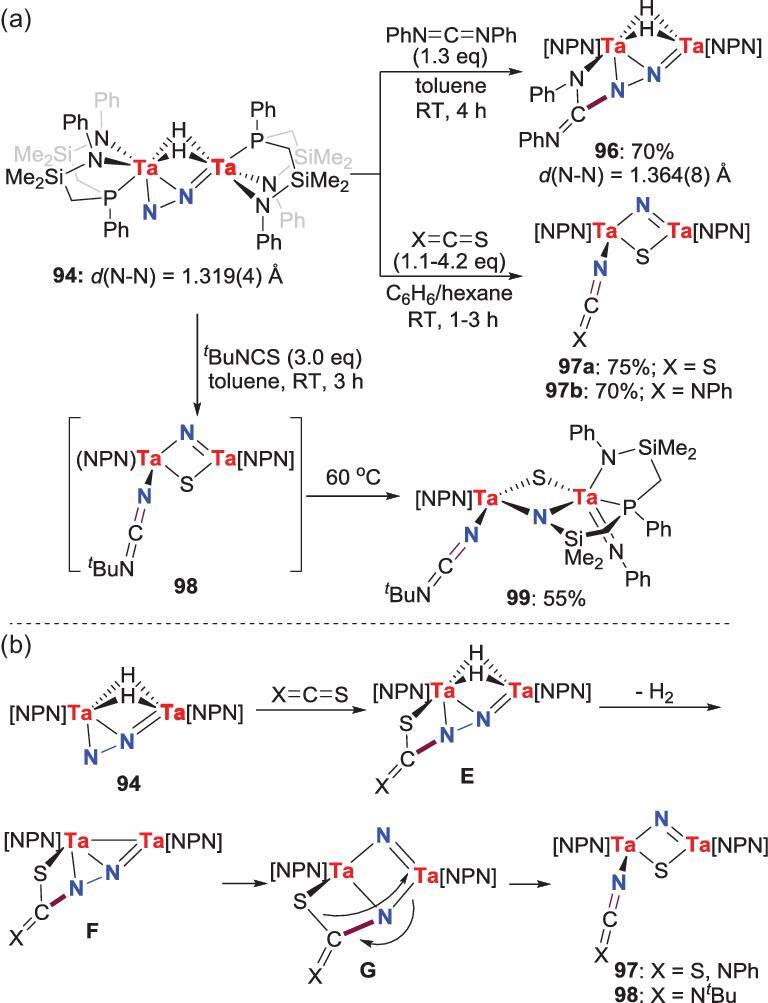
N−C bond formation from the cycloaddition reactions of side-on-end-on N_2_-Ta complexes with heteroatom 1,2-cumulenes. (a) The reaction of N_2_-Ta complex **94** with carbodiimide, carbon disulfite and isothiocyanates. (b) A plausible mechanism for the generation of **97** and **98**.

Besides, this side-on-end-on N_2_-Ta complex **94** was also reported to undergo [2+2] cycloaddition reaction by treating with heteroatom 1,2-cumulenes (Scheme 19a) [59]. For example, the reaction between **94** and *N*,*N*^′^-diphenyl carbodiimide results in the formation of **96**. However, when carbon disulfite or isothiocyanates are added, the *N*-functionalization product **97** is generated concomitant with the N−N bond scission. In the case of *tert*-butyl isothiocyanate (*^t^*BuNCS), the generated intermediate **98** would further undergo N−Si bond formation at elevated temperature to give **99**. As depicted in Scheme 19b, the formation of **97** and **98** can be rationalized by the following mechanism. The initial [2+2] cycloaddition reactions between **94** and the C=S bond of the substrates give intermediate **E**, followed by reductive elimination of H_2_ to provide a transient intermediate **F** that contains a Ta−Ta bond. The Ta−Ta bond in **F** would trigger the N−N bond cleavage to afford the final products.

## N−C BOND FORMATION VIA METAL NITRIDES

The complete reduction of N_2_-M complexes might cleave the N−N bond of the N_2_ ligands to give metal nitrides. In the most terminal metal nitrides, the strong metal−nitrogen bonds result in these nitrides often exhibiting weak nucleophilicity and just reacting with high-energy species such as alkyl triflates and acyl chlorides to assemble N−C bond. However, some bridging nitrides derived from N_2_ can also react with other carbon-based substrates, like MeI and CO, to form N−C bond.

### *N*-alkylation

**Scheme 20.**
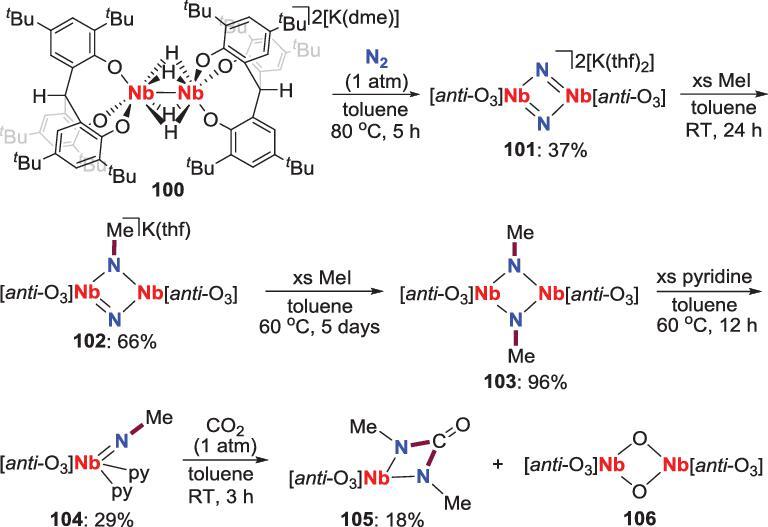
*N*-methylation of Nb-nitride by reaction with MeI.

In 2007, Kawaguchi *et al*. reported the reaction between MeI and bis(*μ*-nitrido) diniobium complex **101**, which is prepared from the tetra(*μ*-hydride) diniobium precursor **100** (Scheme 20) [60]. Stepwise methylation of **101** by MeI yields mono-imido **102** and bi-imido **103**, the latter of which could also react with excess pyridine to give terminal imido **104**, which reacts with CO_2_ to generate **105** and **106** through further N−C bond formation. A plausible mechanism for this process was raised by the authors. A [2+2] cycloaddition of **104** with CO_2_ followed by extrusion of methyl isocyanate (MeNCO) results in the formation of a terminal oxo species that dimerizes to give **106**. Meanwhile, the generated MeNCO would also undergo [2+2] cycloaddition with another molecule of **104** to form **105** [61].

**Scheme 21.**
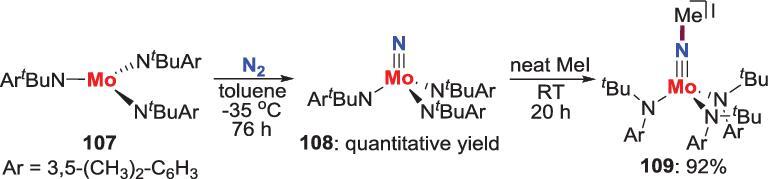
*N*-alkylation of Mo-nitride by reaction with MeI.

Cummins *et al*. found that the terminal molybdenum nitride **108** synthesized from the three coordination Mo(III) complex **107**, undergoes *N*-alkylation by reacting with MeI to provide **109** (Scheme 21) [62].

**Scheme 22.**
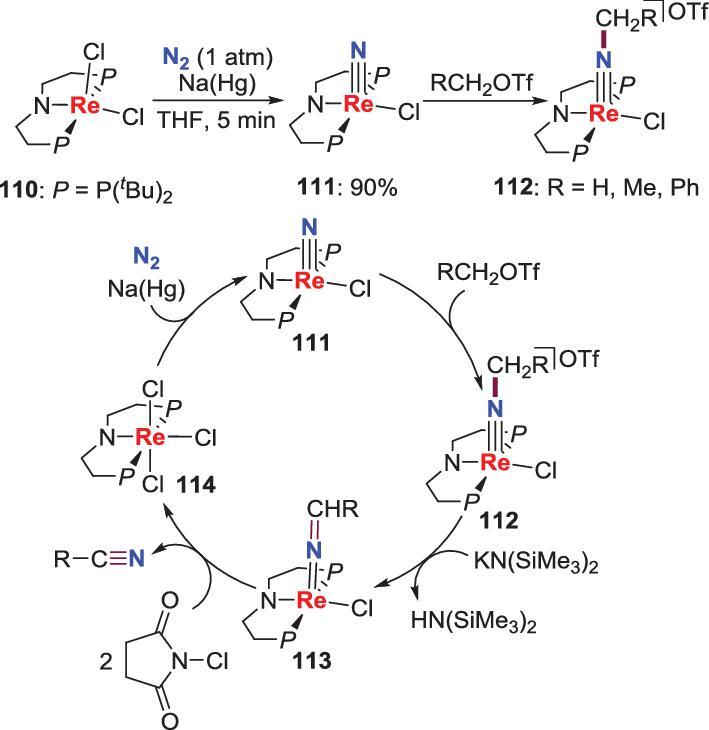
Re-promoted conversion of N_2_ into nitriles via *N*-alkylation of Re-nitride.

Another example of making N−C bond from metal nitrides was fulfilled by Schneider *et al*. in 2016. The reactions between ROTf (R = Me, Et and Bn) and a terminal rhenium nitride **111**, which is prepared from the reduction of the dichloride precursor **110** with sodium amalgam or CoCp^*^_2_, give the *N*-alkylation complexes **112** (Scheme 22) [63–65]. Further studies suggest that the nitriles (RCN) can be liberated by deprotonation and oxidation of **112** via the ketimido intermediates **113**, accompanied by the generation of trichloride complex **114**, which could also be reduced by sodium amalgam to afford the starting material **111**. According to these results, a full synthetic cycle for synthesis of nitriles from N_2_ with moderate isolated yields was established (Scheme 22).

**Scheme 23.**
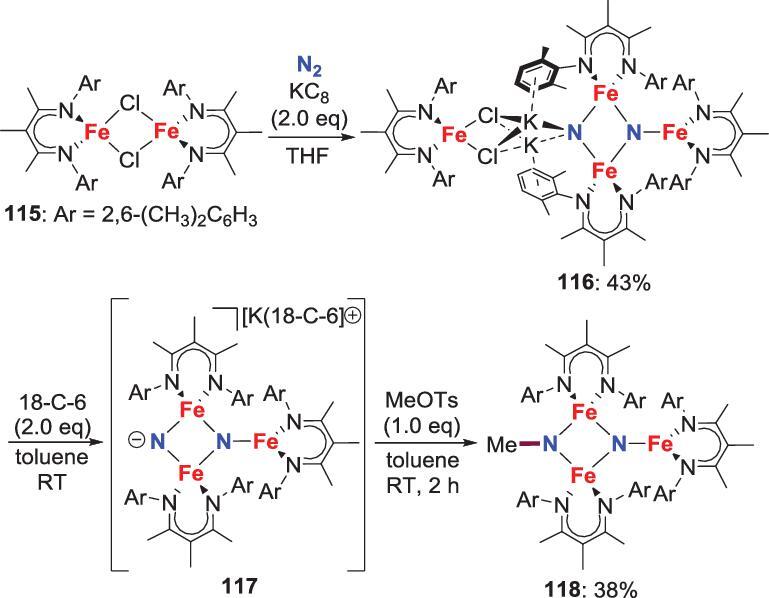
*N*-methylation of Fe-nitride by reaction with MeOTs.

Because the group 8–10 N_2_-derived nitrides are rare, the *N*-functionalization of these nitrides is hardly observed. One exception was reported by Holland *et al*., who employed an unprecedented trinuclear iron nitride **116** to achieve this transformation. This nitride complex **116**, obtained from the reduction of the chloride precursor **115** with precisely equivalent KC_8_ under N_2_ atmosphere, can react with MeOTs and 18-crown-6 (18-C-6) to give the methylimido complex **118** via a presumptive two-coordinate nitride **117** with higher reactivity (Scheme 23) [66].

### *N*-acylation

**Scheme 24.**
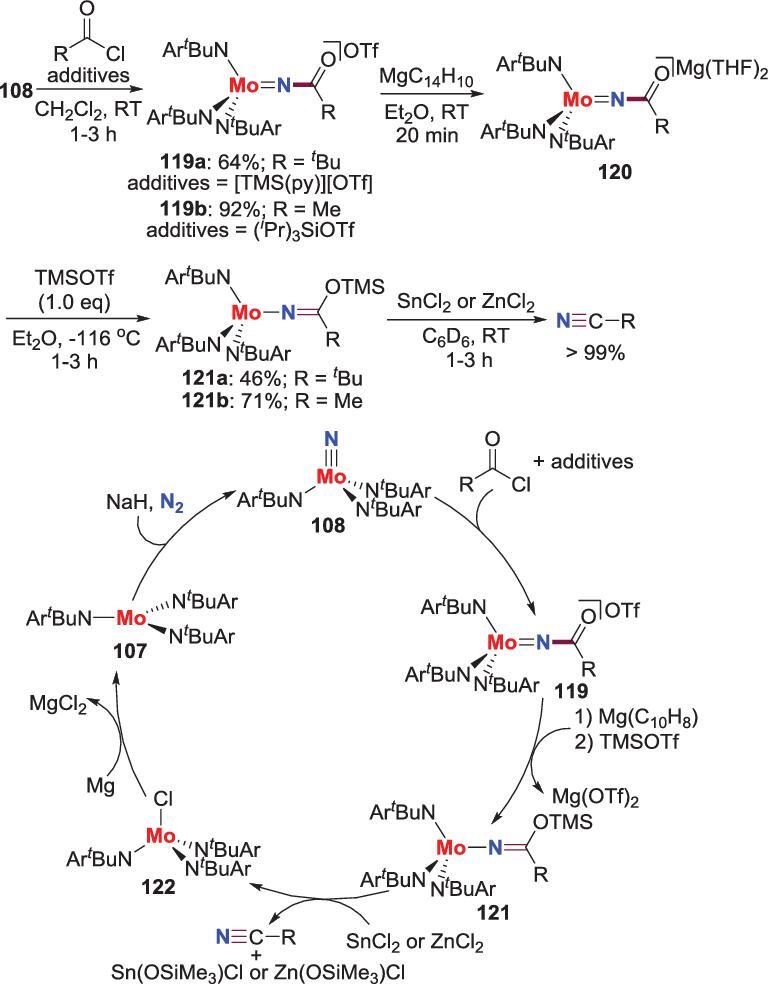
Conversion of N_2_ into nitriles via *N*-acylation of Mo-nitride.

The *N*-acylation of N_2_-derived nitride has also been investigated. For example, the *N*-acylation products **119** are obtained when **108** is treated with acyl chlorides in the presence of additives, such as [TMS(py)][OTf] and *^i^*Pr_3_SiOTf. Furthermore, when the *N*-acylated products **119** reacts with magnesium anthracene (MgC_14_H_10_) and trimethylsilyl triflate (TMSOTf) in one pot, it would be converted to the trimethylsiloxy-substituted ketimide **121** via the intermediates of **120**. Further reaction of **121** with SnCl_2_ or ZnCl_2_ affords the corresponding organic nitriles commitment with the generation of molybdenum chloride complex **122**, a precursor of the trisamide molybdenum complex **107**. In consequence, an efficient synthetic cycle that can directly convert N_2_ to nitrile was accomplished (Scheme 24) [67].

### *N*-acylation/elimination

**Scheme 25.**
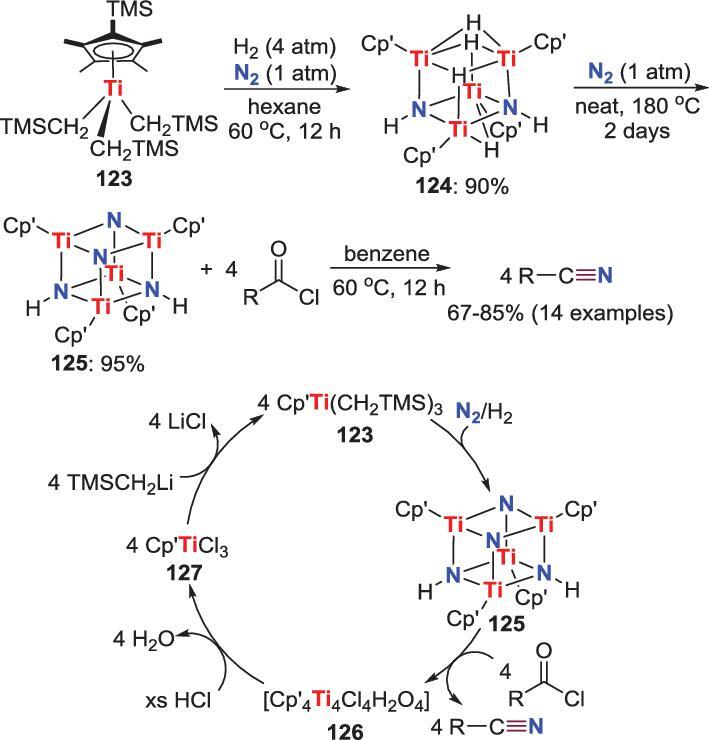
Titanium-promoted direct conversion of N_2_ into nitriles via the reaction between acyl chlorides and Ti-nitride.

In addition to simple *N*-acylated products, the reactions between N_2_-derived metal nitrides and acyl chlorides also afford nitriles proceeded through *N*-acylation and subsequent elimination in formal. Hou *et al*. discovered that the reaction of titanium trialkyl complex **123** with N_2_ and H_2_ results in a novel diimide/tetrahydride complex **124**. This complex can react with N_2_ at elevated temperature to provide a tetranuclear diimide/dinitride complex **125** that can further react with a series of acyl chloride to afford the corresponding nitriles in high yield (Scheme 25) [68]. Based on the experimental and computational results, the authors think that the functionalization of the imide ligands is prior to the nitride groups in these reactions. Furthermore, by treatment of the crude reaction mixture with HCl, the titanium trichloride complex **127** is isolated, which could be easily converted to **123** by reacting with TMSCH_2_Li. Hence, a synthetic cycle of titanium-promoted synthesis of nitriles direct from N_2_ was proposed (Scheme 25).

**Scheme 26.**
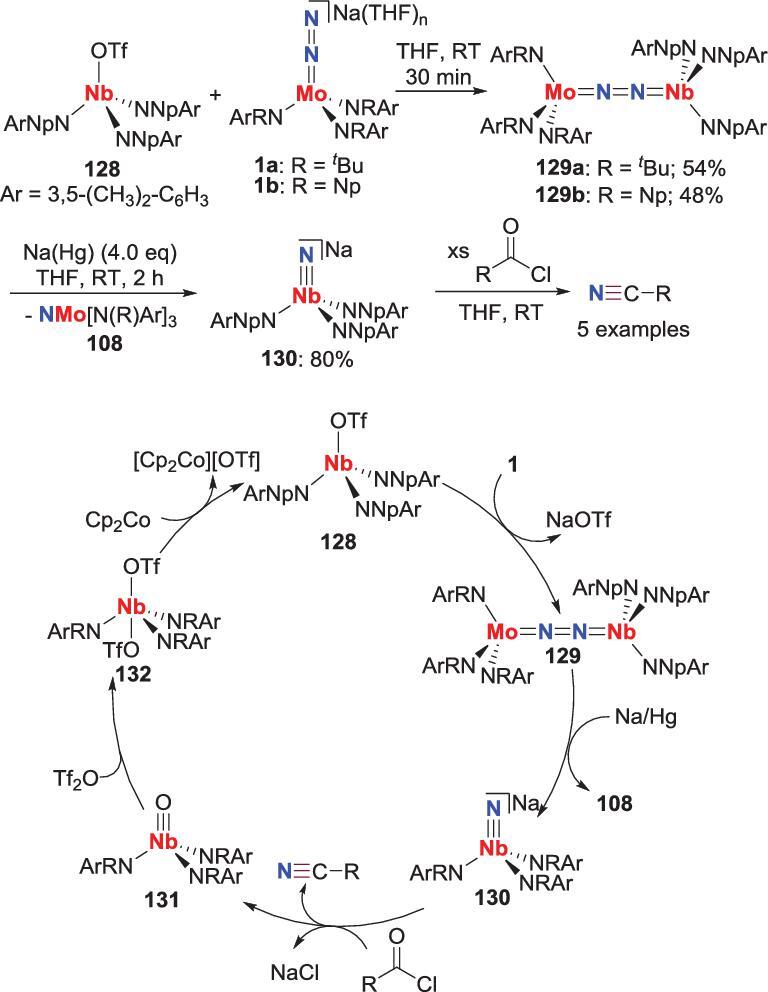
Niobium-promoted direct conversion of N_2_ into nitriles via the reaction between acyl chlorides and Nb-nitride.

Another synthetic cycle for providing organic nitriles from N_2_ was developed by Cummins *et al*. via a niobium nitride intermediate (Scheme 26) [69]. An end-on bridging heterodinuclear N_2_-M complex **129**, prepared from the reaction of the niobium triflate complex **128** and the aforementioned N_2_-Mo complex **1**, could undergo N_2_ ligand scission to form anionic niobium nitride **130** along with the formation of molybdenum nitride **108**, when **129** is treated with sodium amalgam. Treatment of **130** with acyl chloride results in releasing of nitriles accompanied by the generation of the niobium oxo complex **131**. By treating with triflic anhydride, this oxo complex **131** can be converted to a bistriflate complex **132** that could be reduced to the initial compound **128** to finish the cycle.

### Redox-coupled *N*-atom transfer

**Scheme 27.**
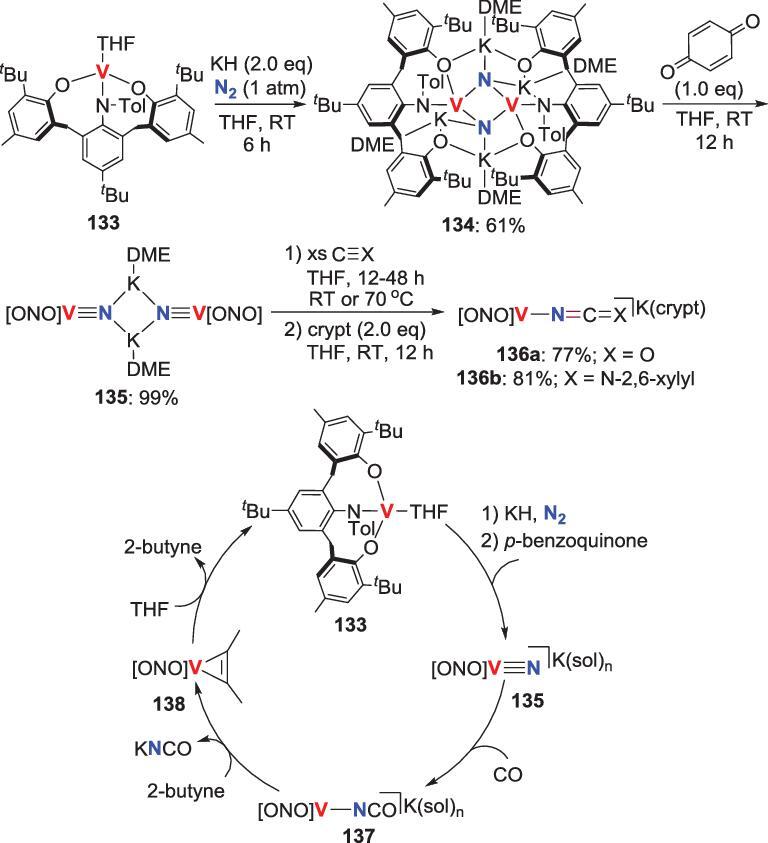
Vanadium-promoted direct conversion of N_2_ into potassium cyanate via the reaction between CO and N_2_-V complex.

In comparison with *N*-alkylation or acylation and subsequent reduction, a more efficient route to transfer the nitride into organic compounds is the transformation of nitride-*N* atom into an incoming substrate with concurrent metal reduction. In 2014, Kawaguchi *et al.* reported the redox-coupled *N*-atom transformation of a V-nitride (Scheme 27) [70]. Reduction of the V(III) complex **133** by KH under N_2_ results in a split of the N_2_ to provide the *μ*-nitride V(IV) complex **134**, which could be oxidized to V(V) nitride compound **135** via reacting with *p*-benzoquinone. When **135** is treated with CO or 2,6-xylylisocyanide in the presence of [2.2.2]-cryptand, the *N*-atom transformation of the substrates is observed concomitant with the formation of **136**. The contact-ion-pair complex **137** could also be isolated from the reaction of **135** and CO. Although the extrusion of the cyanate or carbodiimide ligand in **136** are not easy, the contact-ion-pair **137** readily undergoes ligand exchange with 2-butyne to liberate potassium cyanate (KNCO) with the formation of the alkyne adduct **138**. Additionally, **138** is facilely converted into the starting complex **133** upon dissolving in THF. Hence, a synthetic cycle for direct conversion of N_2_ and CO into KNCO was completed. However, achieving the catalytic process of this synthetic cycle remains elusive due to the incompatibility of the individual steps in this cycle, such as the requirement of solvents in the N−C bond formation step and KNCO releasing step being different.

### *N*-silylation/imido transfer

**Scheme 28.**
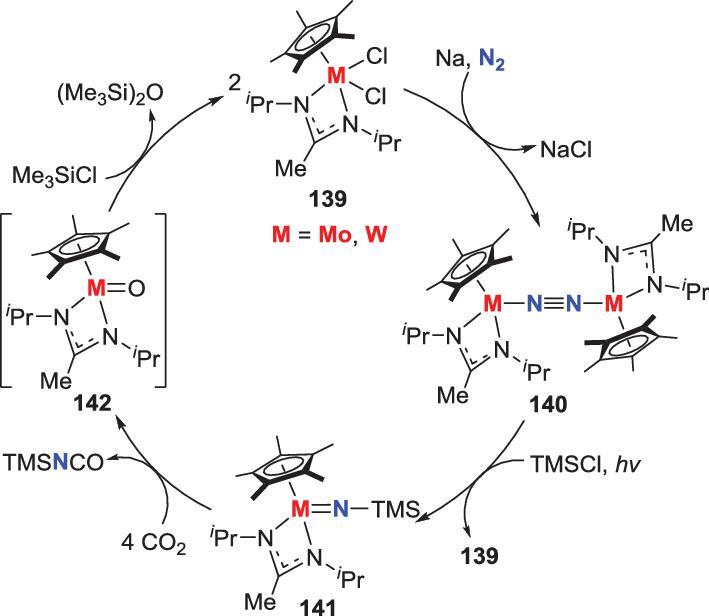
N−C bond formation via the reaction of silyl-imido complexes with CO_2_.

Besides *N*-hydrogenated intermediates, N_2_-derived *N*-silylated complexes are also good precursors to make N−C bond. For instance, a cycle of Mo- and W-promoted synthesis of isocyanates from N_2_ via a silyl-imido intermediate was established (Scheme 28) [71]. Photolysis of the end-on bridging N_2_-Mo, W complexes **140** leads to the generation of nitride intermediates via N−N bond cleavage (*vide infra*), which would be trapped *in situ* by TMSCl to afford silyl-imido complexes **141**. When TMSCl is replaced by Ph_3_SiCl, Me_3_CCl or Me_3_GeCl, the similar reaction could also take place to provide the corresponding imido complexes. Besides, the organic compound TMSNCO could be obtained concomitant with the formation of the mono-nuclear oxo complexes **142** by treatment of **141** with CO_2_. These oxo complexes **142** are known to react with additional TMSCl to regenerate the dichloride complexes **139** that are the precursors of the N_2_-M complexes **140**.

### Metal-ligand cooperative *N*-atom transfer

**Scheme 29.**
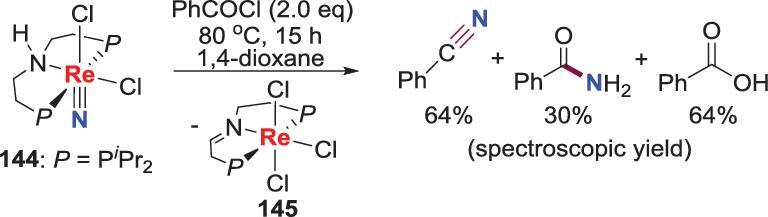
Metal-ligand cooperative *N*-atom transfer of a Re-nitride.

Metal-ligand cooperative *N*-atom transfer is also an efficient strategy because of the avoiding of extra protons and electrons. Recently, a metal-ligand cooperative *N*-atom transfer of a Re-nitride was reported by a cooperative *2 H^+^/2 e^−^* transfer of the pincer ligand (Scheme 29) [72]. This Re-nitride complex **144**, which is generated from photo-promoted cleaving of the end-on bridging binuclear N_2_-Re complex **143** (*vide infra*), could react with benzoyl chloride to afford benzamide (PhCONH_2_), benzonitrile (PhCN) and benzoic acid (PhCOOH) along with the formation of trichloride Re complexes **145**, in which the pincer ligand is oxidized to an imine-type ligand. The producing of PhCN and PhCOOH is caused from the reaction of the initially formed benzamide with excess benzoylchloride in the crude.

### Involvement of photochemistry

**Scheme 30.**
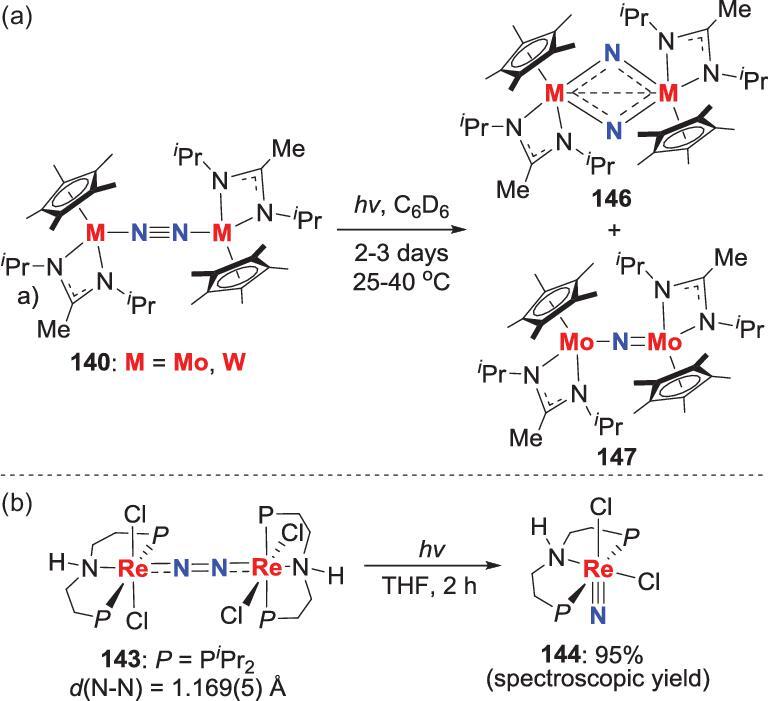
Photolytic cleavage of end-on bridging N_2_-Mo, W and Re complexes into nitrides. (a) Photolytic cleavage of end-on bridging N_2_-Mo, W complexes **140**. (b) Photolytic cleavage of end-on bridging N_2_-Re complexes **143**.

Besides photo-promoted radical generation, another pathway of the photochemistry participating in N_2_ transformation is the direct photolytic splitting of N_2_ ligands into nitrides, which could further engage in *N*-atom transfer. Two examples of this method have been reported. In the first example, irradiation of the above-mentioned end-on bridging N_2_-Mo, W complexes **140** over several days by medium-pressure Hg lamps leads to the generation of two metal nitrides **146** and **147** (Scheme 30a). Furthermore, when **140** are photolyzed in the presence of excess TMSCl, the terminal silylimido complexes **141** are obtained in moderate yield with the formation of dichloride complexes **139** (Scheme 29) [71]. The other example involves the photolysis of an end-on-bridged N_2_-Re complex **143** that has abnormal thermal-stability, to provide the aforementioned Re-nitride **144** (Scheme 30b) [72]. It is noteworthy that the photo source of this reaction could be Xe(Hg) lamp (λ > 305 nm) or a 390 nm LED lamp.

### Involvement of electrochemistry

**Scheme 31.**
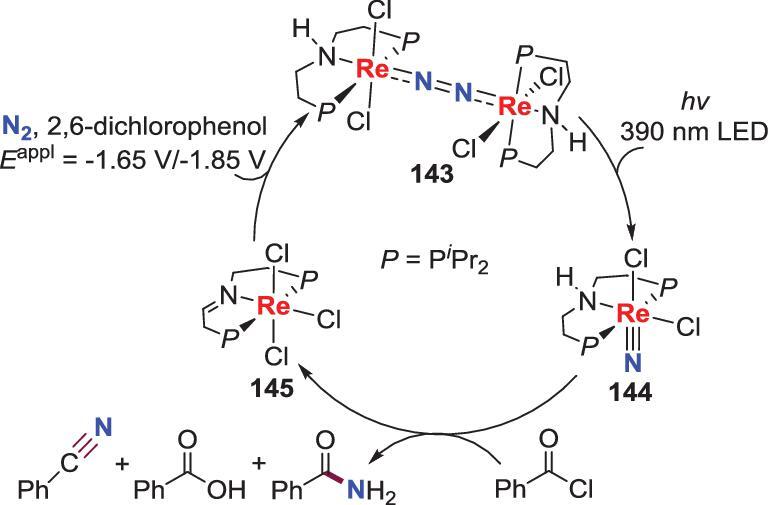
Electrochemical reduction involved synthetic cycle of direct conversion of N_2_ into benzamide and benzonitrile.

Electrochemical N_2_ reduction is an alternative to chemical N_2_ reduction for the synthesis of N_2_-M complexes. This approach has been utilized to regain the N_2_-Re complex **143** to achieve a cycle (Scheme 31) [72]. Schneider *et al.* found that in the controlled potential electrolysis experiment, the trichloride Re complexes **145** formed from the reaction of Re-nitride with PhCOCl, could be converted to the N_2_-Re complex **143** via electrolyzing at *E* = −1.65 V for 8 h in the presence of proton source of 2,6-dichlorophenol (DCP) and subsequently electrolyzing at *E* = −1.85 V for 5 h under N_2_. Thus, a three-step cycle for the synthesis of PhCONH_2_/PhCN from N_2_ was established, in which the creative approaches of metal-ligand cooperation and photo- and electrochemistry were all used.

## N−C BOND FORMATION VIA UNCHARACTERIZED N_2_-M INTERMEDIATES

Compared to the above works, the earlier reports about the conversion of N_2_ into organic compounds were achieved by one-pot reactions of ill-defined N_2_-complexes or their derivatives with carbon-based substrates and followed by hydrolysis. In this section, some examples of this method were introduced briefly.

**Scheme 32.**
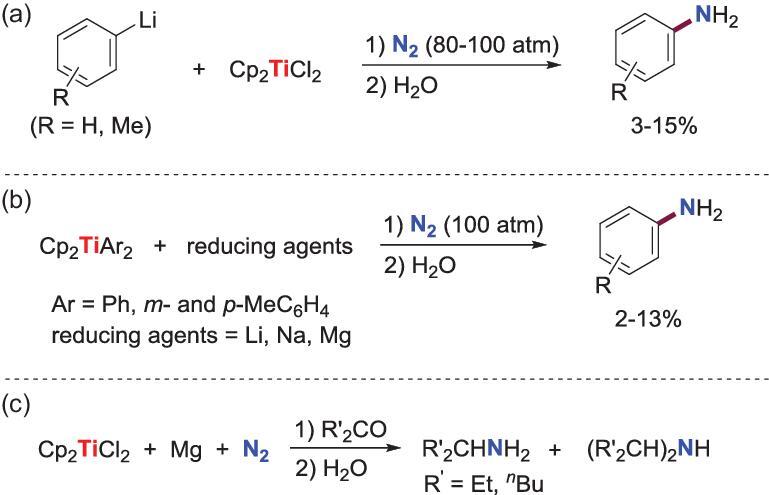
Ti-promoted transformation of N_2_ into amines via ill-defined intermediates. (a) The reaction of Cp_2_TiCl_2_ with aryllithium reagents under N_2_ to afford aromatic amines. (b) The reaction of diaryltitanocenes Cp_2_TiAr_2_ with alkali or alkaline metal under N_2_ to afford aromatic amines. (c) Synthesis of organic amines from the reaction between ketones and a supposed titanium nitride species.

The initial works of transition metal promoted direct conversion of N_2_ into organic compounds were reported more than 50 years ago, when Vol’pin and Shur *et al*. developed two systems for transformation of N_2_ into aromatic amines mediated by titanium species [14,73]. In the first system, several aromatic amines are obtained when Cp_2_TiCl_2_ is treated with excess of aryllithium (aryl = Ph, *m*- and *p*-MeC_6_H_4_) reagents under N_2_ pressure of 80–100 atm and followed by hydrolysis (Scheme 32a). When the aryllithium in these reactions is replaced by alkyllithium reagents, the corresponding alkylamines could not be obtained. The other system, which also gives arylamines by subsequent hydrolysis, involves the reaction of diaryltitanocenes Cp_2_TiAr_2_ (Ar = Ph, *m*- and *p*-MeC_6_H_4_) with alkali or alkaline metal (Li, Na and Mg) and N_2_ (100 atm) (Scheme 32b). Although plenty of effort has been made, the detailed mechanisms of these works are yet unclear.

Meanwhile, a related work was reported in 1970 by van Tamelen and Rudler, who succeeded in the synthesis of organic amines from the reaction between ketones and a supposed titanium nitride species prepared through the reaction of Cp_2_TiCl_2_ with magnesium under N_2_ (Scheme 32c) [74].

**Scheme 33.**
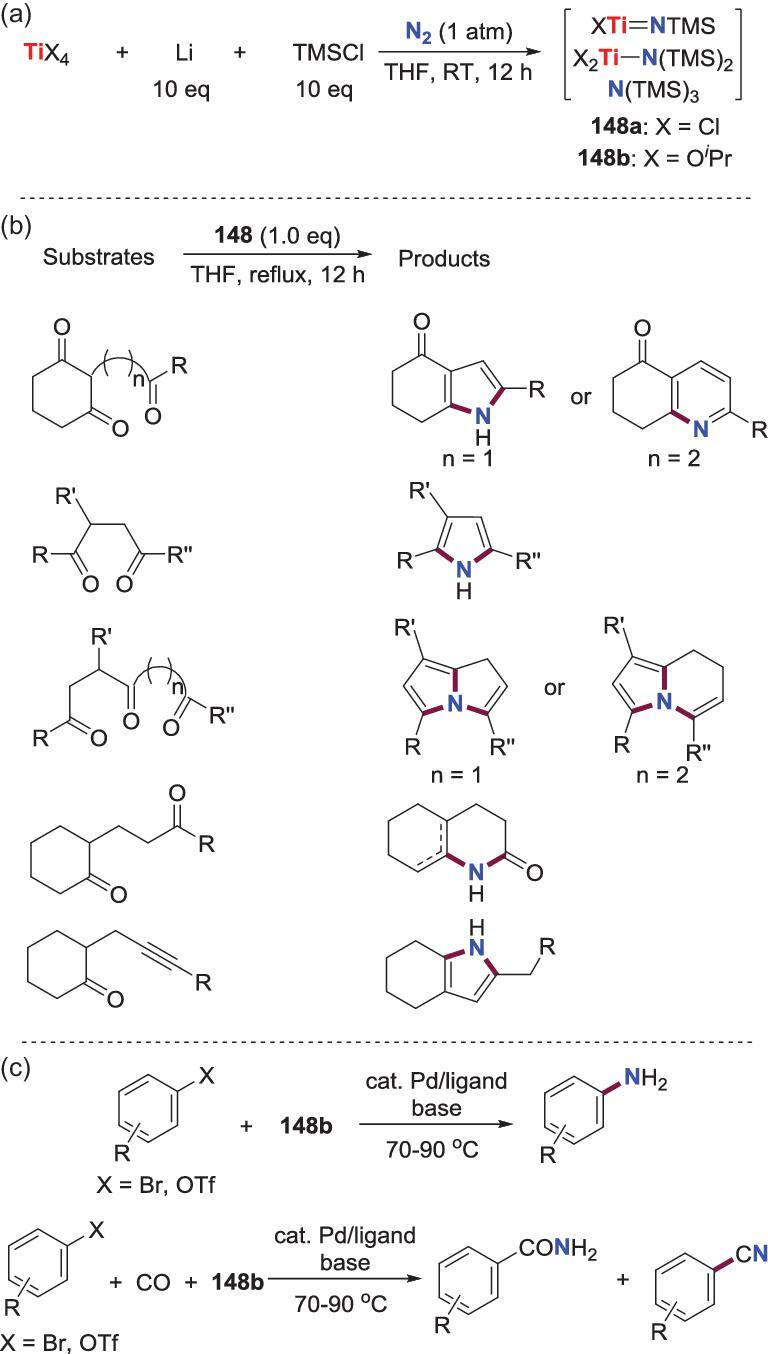
Ti-promoted N−C bond formation via ill-defined *N*-silylation titanium species. (a) Preparation of *N*-silylation titanium complexes **148** from one-pot reaction of TiCl_4_ or Ti(O*^i^*Pr)_4_ with Li and TMSCl under N_2_ or dry air. (b) The reaction between **148** and keto-carbonyl compounds to afford nitric heterocycles. (c) Palladium-catalyzed synthesis of aryl- or allyl- amines and amide derivatives from **148** and aryl or allyl halides in the absence or the presence of CO.

Additionally, Mori *et al*. achieved incorporation of N_2_ into organic compounds via the *N*-silylation titanium complexes **148**, which were prepared from the one-pot reaction of titanium species (TiCl_4_ or Ti(O*^i^*Pr)_4_), Li and TMSCl under N_2_ or dry air (1 atm) [75]. Although the precise components and structures of **148** have not been determined so far, they are considered to contain XTi = NTMS, X_2_TiN(TMS)_2_ (X = Cl, O*^i^*Pr) and N(TMS)_3_ [76–78]. **148** could serve as a nitrogenation reagent to react with a series of keto-carbonyl compounds to provide kinds of nitric heterocycles, such as indole, quinoline, pyrrole, pyrrolizine, lactams and indolizine derivatives. Besides, when **148** is treated with palladium complexes, the transmetalation of *N*-atom from **148** to the palladium center occurs. Hence, palladium-catalyzed synthesis of aryl- or allyl- amines and amide derivatives from aryl or allyl halides and **148** could be fulfilled, in the absence or the presence of CO (Scheme 33).

## CONCLUSION AND OUTLOOK

Direct transformation of N_2_ into *N*-containing organic compounds is of fundamental and practical significance. In the past 60 years, the area of direct incorporation of N_2_ into N−C bond effectuated many great achievements. Relative to the traditional methods of assembling N−C bond via *N*-alkylation of N_2_-M complexes, more atom-efficient approaches, such as cycloaddition, insertion and redox-coupled *N*-atom transfer for making N−C bond have been developed and received more attention in recent years. By the delicate design, some synthetic cycles about direct conversion of N_2_ into organic compounds have also been developed. In these cycles, photo- and electrochemistry are sometimes used to prepare the N_2_-M complexes, cleave the N−N bond or release the final products. However, all of these reactions are stoichiometric and the catalytic system for the direct introduction of N_2_ into organic compounds has not been realized yet. The main factors that prevent these complete synthetic cycles from becoming a catalytic process are the rigorous reaction conditions of the N−C bond formation and *N*-containing organic compounds releasing steps in these cycles, which are incompatible with the preparation steps for N_2_-M complexes. Hence, developing milder systems are imperative. Besides, new reaction types also need to be explored. In this context, we think the following fields can be considered in the future.

### New reaction systems

So far, most N−C bond formation occurs at N_2_-M complexes of group 4–6 transition metals. Exploring other metal promoted N−C bond formation is an attractive topic. Besides, the multi-metal synergistically promoted N_2_ activation and functionalization also need to be studied. Toward this end, the design of new types of ligands should be considered.

### New reaction types

Reductive elimination, an essential step in catalytic amination reactions, has not been found to take place at N_2_-derived *N*-containing transition metal complexes. This process should be explored in future because it provides an efficient approach to assemble N−C bond concomitant with regaining the N_2_-M complexes or their precursors. Additionally, other intriguing reaction modes, such as the [4+2] cycloadditions of N_2_ ligands and insertion of N_2_ into metal**−**carbon bond should also be investigated.

### Polynuclear metal species cooperative N2 scission and functionalization

Stimulated by the previous works on multinuclear Ti, Fe complexes-promoted N_2_ cleavage and N−C bond formation [66,68], the strategy to realize synergistic N_2_-splitting and subsequent functionalization using polynuclear metal complexes should be further explored. Additionally, recent reports on gas-phase polynuclear metal clusters-mediated N_2_ scission and subsequent N−C bond formation deserve further attention [79,80].

### Main group elements promoted N−C bond formation

The recent report about N_2_ reduction by borylenes from Braunschweig *et al*. suggests the potential of boron mediated formation of N−C bond from N_2_ [81]. Besides, some calculation results indicate that the direct reactions of boron or carbene with N_2_ are also permitted in some cases. For example, Li and Schaefer *et al.* designed a new molecular system for nitrogen reduction, involving a 2,3^′^-bipyridine-anchored, end-on-bridging dinitrogen complex of the Me_2_B–BMe_2_ intermediate by theoretical calculations [82]. Zhu *et al.* designed a metal-free dinitrogen activation system based on the boron and NHC carbene system [83]. These results offer inspiration for future work on p-block elements promoting or catalytic conversion of N_2_ into organic compounds.

### Analogue of PCET: lessons from N_2_-to-NH_3_ catalysis system

Very recently, Nishibayashi *et al*. achieved a remarkable N_2_-to-NH_3_ process via a molybdenum-catalysis system. By using the samarium diiodide (SmI_2_) as the reductant and alcohol or water as the proton sources, the total turnover number (TON) of this reaction reaches up to 4350 with 91% yield of NH_3_. Further studies reveal that a proton-coupled electron-transfer (PCET) process, in which O−H bonds in water or alcohols are weakened by coordination to SmI_2_, is the key to this high reactivity [84]. Inspired by this, a similar process of C−X (X = O, Cl, Br, I) bonds coordinated to a relevant reductant to weaken the C−X bonds, which could be named as carbocation-coupled electron-transfer (CCET) should also be studied. This CCET process may offer a path forward for developing catalysis systems that incorporate N_2_ into amines via successive alkylation of a N_2_-derived nitride.

### Photochemistry

Limited complexes capable of N_2_ photoactivation are currently known, and the underlying photophysical and photochemical processes after light absorption are largely unresolved. Light can induce the split of N≡N bond in the M-N_2_ complexes and the resulting nitride complexes are typically reactive. Hence, the following N−C formation reaction should be possible. Besides, the excitation into N−N π^*^ orbitals is also possible, which can lead to a weakened π-bond, and hence a following N−C formation directly from M-N_2_^*^ and carbon-based substrates including CO, CO_2_ might be possible.

### Electrochemistry

Previous work indicates that electrochemical reduction could release final organic compounds along with regeneration of N_2_-M complexes. This stimulates us to create an electrochemical reduction system, in which the electrolyzation step is compatible with the N−C bond formation step. Besides, the recent report of N_2_ and CO_2_ coupling to produce urea, which was conducted by an electrocatalyst consisting of PdCu alloy nanoparticles on TiO_2_ nanosheets, suggests that designing a new solid catalyst to incorporate N_2_ into high-value *N*-containing product beyond NH_3_ should also be attractive [85].

### Heterogeneous catalysis systems

Although the industrial Haber-Bosch process produces NH_3_ over the surface of heterogeneous solid-state catalysts, a similar process, in which a heterogeneous catalyst catalyzes or promotes direct transformation of N_2_ into organic compounds, has not been reported in literature. Development of the new systems, where the merits of the homogeneous molecular systems and the heterogeneous systems are rationally combined, is a promising approach toward the goal.

### Via *N*-protonation or silylation intermediates

The strategy of conversion of N_2_ into organic compounds via the *N*-silylation and *N*-protonation complexes can be further extended. As an example, converting the less active M-N_2_ complexes into M-N−Si/H species *in situ* followed by catalytic reaction with carbon-based substrates, might result in various valuable organic compounds being synthesized.

In a word, with combined efforts from cross-disciplines the dream of direct catalytic and efficient conversion of N_2_ into *N*-containing organic compounds under mild conditions is believed to be attainable in the future.
